# Hypoxic Incubation Conditions for Optimized Manufacture of Tenocyte-Based Active Pharmaceutical Ingredients of Homologous Standardized Transplant Products in Tendon Regenerative Medicine

**DOI:** 10.3390/cells10112872

**Published:** 2021-10-25

**Authors:** Annick Jeannerat, Cédric Peneveyre, Florence Armand, Diego Chiappe, Romain Hamelin, Corinne Scaletta, Nathalie Hirt-Burri, Anthony de Buys Roessingh, Wassim Raffoul, Lee Ann Applegate, Alexis Laurent

**Affiliations:** 1 Applied Research Department, LAM Biotechnologies SA, CH-1066 Épalinges, Switzerland; annick.jeannerat@lambiotechnologies.com (A.J.); cedric.peneveyre@lambiotechnologies.com (C.P.); 2 Proteomics Core Facility and Technology Platform, Ecole Polytechnique Fédérale de Lausanne, CH-1015 Lausanne, Switzerland; florence.armand@epfl.ch (F.A.); diego.chiappe@epfl.ch (D.C.); romain.hamelin@epfl.ch (R.H.); 3 Regenerative Therapy Unit, Lausanne University Hospital, University of Lausanne, CH-1066 Épalinges, Switzerland; corinne.scaletta@chuv.ch (C.S.); nathalie.burri@chuv.ch (N.H.-B.); lee.laurent-applegate@chuv.ch (L.A.A.); 4 Children and Adolescent Surgery Service, Lausanne University Hospital, University of Lausanne, CH-1011 Lausanne, Switzerland; anthony.debuys-roessingh@chuv.ch; 5 Lausanne Burn Center, Lausanne University Hospital, University of Lausanne, CH-1011 Lausanne, Switzerland; wassim.raffoul@chuv.ch; 6 Plastic, Reconstructive, and Hand Surgery Service, Lausanne University Hospital, University of Lausanne, CH-1011 Lausanne, Switzerland; 7 Center for Applied Biotechnology and Molecular Medicine, University of Zurich, CH-8057 Zurich, Switzerland; 8 Oxford OSCAR Suzhou Center, Oxford University, Suzhou 215123, China; 9 Manufacturing Department, TEC-PHARMA SA, CH-1038 Bercher, Switzerland

**Keywords:** active pharmaceutical ingredients, cell banking, cell manufacture, hypoxia, human progenitor tenocytes, optimization, proteomics, regenerative medicine, standardized transplants, tendon affections

## Abstract

Human fetal progenitor tenocytes (hFPT) produced in defined cell bank systems have recently been characterized and qualified as potential therapeutic cell sources in tendon regenerative medicine. In view of further developing the manufacture processes of such cell-based active pharmaceutical ingredients (API), the effects of hypoxic in vitro culture expansion on key cellular characteristics or process parameters were evaluated. To this end, multiple aspects were comparatively assessed in normoxic incubation (i.e., 5% CO_2_ and 21% O_2_, standard conditions) or in hypoxic incubation (i.e., 5% CO_2_ and 2% O_2_, optimized conditions). Experimentally investigated parameters and endpoints included cellular proliferation, cellular morphology and size distribution, cell surface marker panels, cell susceptibility toward adipogenic and osteogenic induction, while relative protein expression levels were analyzed by quantitative mass spectrometry. The results outlined conserved critical cellular characteristics (i.e., cell surface marker panels, cellular phenotype under chemical induction) and modified key cellular parameters (i.e., cell size distribution, endpoint cell yields, matrix protein contents) potentially procuring tangible benefits for next-generation cell manufacturing workflows. Specific proteomic analyses further shed some light on the cellular effects of hypoxia, potentially orienting further hFPT processing for cell-based, cell-free API manufacture. Overall, this study indicated that hypoxic incubation impacts specific hFPT key properties while preserving critical quality attributes (i.e., as compared to normoxic incubation), enabling efficient manufacture of tenocyte-based APIs for homologous standardized transplant products.

## 1. Introduction

Human fetal progenitor tenocytes (hFPT) produced in vitro under defined multi-tiered cell bank systems have recently been characterized (i.e., in vitro and in preclinical models) and qualified as potential therapeutic cell sources in tendon regenerative medicine [1,2,3,4,5]. Potential therapeutic uses of such novel tenocyte-based therapies or products include the management of local inflammation-mediated tissue degeneration, partial tendon ruptures, or volumetric tissue defects related to traumatic injuries. Therein, allogenic applications of homologous cell-based or cell-derived preparations are considered based on the intrinsic advantages of the biological active pharmaceutical ingredients (API) of interest, such as technical simplicity of manufacture, a defined and stable tissue-specific phenotype in vitro, extensive stability, and the verifiable absence of immunogenic or tumorigenic risk factors [1,2,5]. The appropriate conjugation of hFPTs with suitable delivery vehicles (e.g., hyaluronan-based hydrogels) or tissue engineering scaffolds (e.g., synthetic polymer-based constructs, decellularized human or equine tendon tissues) represents a cornerstone in tangible therapeutic combination product development [2,4,6].

Within the establishment of scalable hFPT (i.e., cellular API) manufacturing processes, multiple parameters require appropriate technical optimization and validation phases in order to comply with the current systematic requirements of a risk-based, process-oriented, and quality-driven production approach [5]. Key or critical workflow parameters to be taken into consideration during API manufacturing process design and validation phases include specified contact-process consumables (e.g., proliferation medium, culture vessels, dissociation reagents, and cryopreservation medium), the technical specifications for hFPT culture-expansion (e.g., cell seeding and harvest procedures, media exchanges, total culture periods), as well as incubation parameters (e.g., incubator temperature, humidity levels, gaseous mix composition) [7,8]. The adequate selection of consumable options and process parameters are paramount to ensuring the consistency in both quality and safety of the manufactured cells, to be used as intermediary or bulk raw materials (e.g., for further derivation of lysates or sub-cellular vesicles) or as an API in its final form for standardized transplant development (e.g., extemporaneous thawing and seeding of viable hFPTs on suitable implantable scaffolds).

In view of eventual clinical translation, robust and sustainable hFPT manufacturing processes have already been technically optimized and established in traditionally used and defined normoxic cell culture conditions (i.e., 21% O_2_, 5% CO_2_) following a single organ donation [1,5]. However, healthy human tendons are constituted by poorly vascularized tissues, physiologically exposed to oxygen levels far below 21% O_2_, contrasting with standard and artificial in vitro normoxic culture environments. From a manufacturing standpoint, switching the hFPT in vitro culture parameters (i.e., gaseous mix composition) to an O_2_ level closer to physiological conditions may drastically influence the key cellular characteristics of the biological APIs of interest, to be potentially applied for future therapeutic uses. Provided that such modified production workflows do not negatively influence the quality and safety of cultured cell populations, such manufacturing settings could potentially be of interest for the homologous treatment of tendon tissues with allogenic hFPTs, by aligning the API production environment parameters with the corresponding in vivo tissular characteristics.

Hypoxic cell culture or hypoxic preconditioning of therapeutic cellular materials has been extensively studied, notably in the field of stem cells, in view of enhancing relevant cellular properties and functions or for optimizing the production of cell-free APIs and substances (e.g., exosomes, secretome fractions) [9,10,11,12,13,14,15,16,17,18,19]. The cultivation of cells in relatively low (i.e., as compared to normal atmospheric oxygen levels) O_2_ partial pressures (e.g., 1–5% O_2_) is known to modify the genetic expression patterns of various cell types, which intrinsically possess adaptation mechanisms to ischemia or hypoxia [20,21,22]. The major documented implications of these cellular responses to hypoxia include modifications in metabolic activities, in extracellular matrix (ECM) production and organization, or in the production of sub-cellular vesicles such as exosomes [17,19,23,24,25,26,27]. From the technical viewpoint of therapeutic cellular API manufacturing, tangible advantages have been reported about the use of hypoxic cell culture or cell conditioning, such as the enhancement of cell proliferation, in vitro lifespan, function, potency, survival, and in vivo persistence [28,29,30,31,32,33,34,35,36]. A specific investigation of cellular responses to hypoxia at a transcriptomic or proteomic level enables further general elucidation of the mediators of said specific responses (e.g., HIF-1 expression modulation) and investigation of clinically relevant mechanisms of disease [37,38,39,40,41,42]. Finally, reported evidence suggests that the defined hypoxic in vitro culture of therapeutic cellular materials procures advantages in tissue engineering construct fabrication and some clinical benefits following specific patient treatments [43,44,45,46,47].

The aim of the present study was therefore to comparatively assess the effects of hypoxic in vitro cell culture conditions (i.e., hereafter defined as 5% CO_2_ and 2% O_2_) vs. the standard gaseous mix (i.e., 5% CO_2_ and 21% O_2_) used in the manufacture of hFPTs. Multiple endpoints and parameters were taken into account at cellular and proteomic levels, in order to determine if hypoxic in vitro culture resulted in technically and qualitatively significant differences with regard to potential next-generation cell manufacturing workflows of tenocyte-based APIs for homologous standardized transplant products.

## 2. Materials and Methods

### 2.1. Primary Cell Sources and In Vitro Culture Conditions

The progenitor tenocyte source used for the presented in vitro experiments consisted of banked primary human diploid cells. Cryopreserved hFPTs (i.e., FE002-Ten cell type), enzymatically isolated under the Swiss progenitor cell transplantation program, were used as the main API source in the present study [48]. Therefore, hFPTs were serially culture-expanded in T75 cell culture flasks (75 cm^2^, TPP, Trasadingen, Switzerland) in complete growth medium (CM-FBS) composed of high-glucose DMEM (Gibco™, ThermoFisher Scientific, Waltham, MA, USA) supplemented with 2 mM L-glutamine (Gibco™, USA) and 10% *v/v* fetal bovine serum (FBS, Sigma-Aldrich^®^, St. Louis, MI, USA). Cells were seeded at a relative viable density of 1.5 × 10^3^ cells/cm^2^ and maintained in culture until monolayers attained confluency, with medium exchange procedures performed twice weekly. The cultures were parallelly maintained in humidified incubators at 37 °C under two distinct gaseous mix compositions, namely the “normoxia condition” (i.e., 5% CO_2_ and 21% O_2_) and the “hypoxia condition” (i.e., 5% CO_2_ and 2% O_2_). hFPTs were harvested and used between passages 5 and 8. The cells were continuously cultured until reaching confluency before being used for experiments in both specified conditions (i.e., 14 ± 2 days in T75 flasks).

Cryopreserved primary adipose-derived mesenchymal stem cells (i.e., ASC-F cell type) were purchased from ZenBio Inc. (Durham, NC, USA). The cells were initiated according to the manufacturer’s specifications, seeded at a relative viable density of 3.0 × 10^3^ cells/cm^2^ in T75 flasks, and expanded in specific growth medium (CM-HPL) composed of high-glucose DMEM (Gibco™, USA) supplemented with 5% *v/v* human platelet lysate (HPL, Stemulate^®^, Sexton Biotechnologies, Indianapolis, IN, USA). Cultures were maintained in normoxia conditions until monolayers attained confluency, with medium exchange procedures performed twice weekly. ASCs were harvested and used for experiments at passage 4.

Cryopreserved primary tenocytes isolated from an adult patient (i.e., Ad-Ten-001 cell type, derived from an ablated digit flexor tendon of a 74-year-old human female) were obtained from the Biobank of the Department of Musculoskeletal Medicine at the Centre hospitalier universitaire vaudois (Lausanne, Switzerland). The cells were expanded in normoxia and used for experiments between passages 5 and 8.

### 2.2. Timecourse of HIF-1 Induction with Western Blotting

hFPTs were harvested from confluent normoxic T75 flasks and seeded at a relative viable density of 1.5 × 10^3^ cells/cm^2^ in 6 cm diameter Petri dishes (Falcon^®^, Corning, New York, NY, USA), and further incubated in normoxic culture conditions until cell monolayers reached 80% confluency. The culture vessels were then transferred in hypoxic incubation conditions. At various specified timepoints following transfer of the cultures to hypoxic conditions (i.e., after 3, 6, 9, 24, 32, 49, 56, 72, and 144 h, respectively), individual culture vessels were sequentially removed from the hypoxia incubator and immediately processed. At the time of harvest, the dishes were transferred on ice, and cell monolayers were washed twice with ice-cold phosphate-buffered saline (PBS, Bichsel AG, Unterseen, Switzerland) before the addition of RIPA lysis buffer (Abcam, Cambridge, UK) supplemented with 1 × protease inhibitors (Promega Corporation, Madison, WI, USA). The cellular materials were then collected with a cell scrapper and transferred to 1.5 mL tubes (Eppendorf, Hamburg, Germany), which were incubated for 15 min on ice with intermittent vortexing. Cell lysates were then centrifuged at 10^3^× *g* for 5 min at ambient temperature and supernatants were transferred to new 1.5 mL test tubes. The total protein contents in the samples were determined using the Pierce™ BCA protein assay (ThermoFisher Scientific, USA). Quantities of 5 µg of total protein/sample were subsequently separated by electrophoresis on NuPAGE™ 4–12% Bis-tris polyacrylamide gels (ThermoFisher Scientific, USA), and were transferred onto nitrocellulose membranes (Amersham™ Protran™, Cytiva, Marlborough, MA, USA). The membranes were blocked with PBS-Tween^®^ 20 at 0.05% *v/v* (Applichem, Darmstadt, Germany) supplemented with 5% *w/v* skim milk (Régilait, Saint-Martin-Belle-Roche, France) for 15 min, and were further incubated overnight at 4 °C with primary antibodies, namely anti-HIF-1α (ref. 610958, BD Biosciences, Franklin Lakes, NJ, USA) or anti-actin (ref. PA1-21167, ThermoFisher Scientific, USA). The membranes were then washed thrice with PBS-Tween^®^ 20 buffer and incubated for 1 h at ambient temperature with corresponding secondary anti-mouse HRP antibodies (ref. 31432, ThermoFisher Scientific, USA) or anti-rabbit HRP antibodies (ref. 20403, Biotium Inc., Fremont, CA, USA). The membranes were finally developed using the chemiluminescence detection system ECL™ Prime (Cytiva, USA) on a Uvitec Mini HD9 gel imager (Cleaver Scientific, Rugby, UK). Quantitative data of relative HIF-1α detection (i.e., expression ratios) were obtained by ImageJ (NIH, Bethesda, MD, USA) analysis of gel images.

### 2.3. hFPT Comparative Proliferation Assays

hFPTs were harvested from confluent normoxic T75 flasks using TrypLE™ 1 × dissociation reagent (Gibco™, USA). Cells were enumerated and seeded in triplicate at a relative viable density of 1.5 × 10^3^ cells/cm^2^ in 24-well cell culture microplates using 1.0 mL of CM-FBS. Cultures were incubated and parallelly maintained in both normoxia and hypoxia conditions for up to 7 days. The growth medium was exchanged on day 3 following culture initiation (i.e., day 0). On days 5, 6, and 7 following culture initiation, the cells were harvested as described previously and enumerated on C-Chip Neubauer hemocytometers (NanoEnTek, Seoul, Korea).

### 2.4. hFPT Comparative Surface Marker Panel Characterization by Flow Cytometry

Cell surface marker panel characterization was comparatively performed on hFPTs (i.e., cells at the same passage levels, for two sequential passages) that were expanded for 14 ± 2 days in normoxic and hypoxic culture conditions, respectively, in order to determine if the composition of the gaseous mix induced differential expression of the clusters of differentiation (CD) of interest. Cells were harvested as described previously from confluent T75 flasks (TPP, Switzerland), and maintained in normoxic and hypoxic conditions, before total and viable cell counts were determined on hemocytometers (NanoEnTek, Korea) using Trypan Blue exclusion dye (Sigma-Aldrich^®^, USA). Harvested cells were then suspended at a final total density of 2 × 10^6^ cells/mL in FACS buffer composed of PBS (Bichsel, Switzerland) supplemented with 2% *v/v* FBS (Sigma-Aldrich^®^, USA). Selected cell surface markers were detected by flow cytometry after appropriate incubation of samples (i.e., 10^5^ cells/sample, 1 h incubation) with specific primary anti-human antibodies directly coupled with fluorescein isothiocyanate (FITC) or phycoerythrin (PE) fluorophores (i.e., 1–5 µL of antibody solution/sample). Selected cell surface markers and corresponding antibodies were as follows:

- CD90 (PE Mouse Anti-Human CD90, BD Biosciences, USA, ref. 561970);
- CD73 (PE Mouse Anti-Human CD73, BD Biosciences, USA, ref. 550257);
- CD105 (PE Mouse Anti-Human CD105, BD Biosciences, USA, ref. 560839);
- CD19 (PE Mouse Anti-Human CD19, BD Biosciences, USA, ref. 555413);
- CD34 (PE Mouse Anti-Human CD34, BD Biosciences, USA, ref. 560941);
- CD14 (FITC Mouse Anti-Human CD14, BD Biosciences, USA, ref. 555397);
- CD45 (FITC Mouse Anti-Human CD45, BD Biosciences, USA, ref. 560976);
- CD44 (PE Mouse Anti-Human CD44, BD Biosciences, USA, ref. 550989);
- CD26 (PE Mouse Anti-Human CD26, BD Biosciences, USA, ref. 555437);
- CD166 (PE Mouse Anti-Human CD166, BD Biosciences, USA, ref. 559263);
- MHC class I HLA-ABC (PE Mouse Anti-Human HLA-ABC, BD Biosciences, USA, ref. 560964);
- MHC class II HLA-DR,-DP,-DQ (FITC Mouse Anti-Human HLA-DR,-DP,-DQ, BD Biosciences, USA, ref. 555558);
- IgG1 isotype (PE Mouse IgG1, κ Isotype Control, BD Biosciences, USA, ref. 555749);
- IgG1 isotype (FITC Mouse IgG1, κ Isotype Control, BD Biosciences, USA, ref. 554679);
- IgG2a isotype (FITC Mouse IgG2a, κ Isotype Control, BD Biosciences, USA, ref. 555573).

Appropriate isotype controls were used to determine nonspecific antibody binding. Samples were run on a BD Accuri™ C6 Plus FACS system (BD Biosciences, USA), with a target of 5 × 10^4^ events in the population gate, using 150 µL volumes for each sample. Data analysis and presentation was performed using the BD Accuri™ C6 software (BD Biosciences, USA).

### 2.5. hFPT Comparative Phenotypic Stability in Chemical Adipogenesis and Osteogenesis Induction Models

hFPT differentiation potentials under adipogenic and osteogenic culture conditions under normoxia and hypoxia, respectively, were comparatively evaluated, as described below, in order to determine the potential impacts of hypoxia on specific progenitor cell potency or phenotypic stability. ASC-F cells were used as positive differentiation controls.

#### 2.5.1. Comparative Chemical Adipogenic Differentiation Assay

hFPTs and ASC-F cells were seeded at a relative viable density of 1.5 × 10^3^ cells/cm^2^ in multiple 12-well cell culture microplates (Falcon^®^, USA) in CM-FBS or CM-HPL growth medium, respectively. Cultures were appropriately maintained in both incubation conditions (i.e., normoxia and hypoxia) until cell monolayers attained 80% confluency (i.e., 6 ± 2 days). Thereafter, the specific culture medium was replaced with a specific adipogenic induction medium, composed of high-glucose DMEM (Gibco™, USA) supplemented with 10% *v/v* FBS (Sigma-Aldrich^®^, USA), 2 mM L-glutamine (Gibco™, USA), ITS 1 × (i.e., final concentrations of 10 mg/L insulin, 5.5 mg/L transferrin, and 6.7 µg/L selenious acid, Corning^®^, USA), 1 µM dexamethasone (Acros Organics™, ThermoFischer Scientific, USA), 100 µM indomethacin (Acros Organics™, USA), and 100 µM IBMX (Alfa Aesar™, ThermoFisher Scientific, USA). The induction medium was exchanged twice weekly for a period of 14 days for the different cell types and in both incubation conditions. At the end of the induction period, the cells were fixed with a 4% formalin solution and appropriately stained with Oil Red-O (Sigma-Aldrich^®^, USA) for revelation of lipid droplet accumulation. Following staining, assay microplates were photographed on an Olympus CX30 contrast phase microscope (Olympus Corporation, Shinjuku, Tokyo, Japan).

#### 2.5.2. Comparative Chemical Osteogenic Differentiation Assay

Pre-treatment of assay microplates (Falcon^®^, USA) was performed with the coating of multiple 12-well cell culture microplate wells using 50 µg/mL rat tail collagen I (Corning^®^, USA). hFPTs and ASC-F cells were then seeded at a relative viable density of 1.5 × 10^3^ cells/cm^2^ in the microplates in FBS- or HPL-supplemented growth medium, as described previously, respectively. Cultures were appropriately maintained in both incubation conditions (i.e., normoxia and hypoxia) until cell monolayers attained 80% confluency (i.e., 6 ± 2 days). Thereafter, the specific culture medium was replaced with a specific osteogenic induction medium, composed of high-glucose DMEM (Gibco™, USA) supplemented with 5% *v/v* HPL (Stemulate^®^, USA), 80 µg/mL VitCp (Sigma-Aldrich^®^, USA), 5 mM β-glycerophosphate (Sigma-Aldrich^®^, USA), and 100 nM dexamethasone (Acros Organics™, USA). The induction medium was exchanged twice weekly for a period of 21 days for the different cell types and in both incubation conditions. At the end of the induction period, the cells were either fixed with a 4% formalin solution and stained with a classical Von Kossa staining procedure (Sigma-Aldrich^®^, USA) or were fixed with 70% ethanol and stained with Alizarin Red stain (Sigma-Aldrich^®^, USA) for revelation of mineralized matrix accumulation. Following staining, assay plates were photographed as described previously.

### 2.6. hFPT Comparative Proteomic Analysis by Quantitative Mass Spectrometry

#### 2.6.1. Sample Preparation for LC-MS/MS

hFPTs were harvested from normoxic and hypoxic confluent T75 flasks (TPP, Switzerland) after 14 ± 2 days of culture. Adult tenocyte cultures that were initiated and maintained in normoxia were processed in the same way. Harvested cells were respectively split in 2 × 10^6^ cell aliquots and were subsequently washed in PBS (Bichsel, Switzerland). Cell lysis was chemically performed in a pH 8.5 lysis buffer composed of LC-MS grade water (Pierce™, ThermoFisher Scientific, USA) supplemented with 2% SDS (Sigma-Aldrich^®^, USA), 100 mM Hepes (Sigma-Aldrich^®^, USA), and 25 mM DTT (Sigma-Aldrich^®^, USA). Following the cell lysis step, the samples were sonicated on ice and subsequently heated at 56 °C for 20 min. The total protein contents in the samples were then determined using a Pierce™ BCA Protein Assay Kit–Reducing Agent Compatible (ThermoFisher Scientific, USA), according to the manufacturer’s protocol and specifications. The total protein sample concentration was adjusted at 1 µg/mL by appropriate dilution before the sample digestion steps.

Samples were then digested using the filter-aided sample preparation (FASP) protocol with minor modifications [49]. Protein lysates were resuspended in 200 µL of 8 M urea (Sigma-Aldrich^®^, USA), 100 mM Tris-HCl pH 8 (Sigma-Aldrich^®^, USA), and deposited on top of Microcon^®^-30K devices (Merck-Millipore^®^, Burlington, MA, USA). Samples were centrifuged at 9391× *g* at 20 °C for 30 min. All subsequent centrifugation steps were performed using the same conditions. An additional 200 µL of 8 M urea and 100 mM Tris-HCl were added, and the devices were centrifuged again. Reduction was performed by adding 100 µL of 10 mM TCEP (Sigma-Aldrich^®^, USA) in 8 M urea, 100 mM Tris-HCl on top of the filters, followed by a 60 min incubation period at 37 °C with gentle shaking and protection from light. The reduction solution was removed by centrifugation and filters were washed with 200 µL of 8 M urea and 100 mM Tris-HCl. After removal of the washing solution by centrifugation, alkylation was performed by adding 100 µL of 40 mM chloroacetamide (Sigma-Aldrich^®^, USA) in 8 M urea and 100 mM Tris-HCl, and incubating the filters at 37 °C for 45 min with gentle shaking and protection from light. The alkylation solution was removed by centrifugation and another washing/centrifugation step with 200 µL of 8 M urea and 100 mM Tris-HCl was performed. This urea buffer washing step was repeated twice, followed by three additional washing steps with 100 µL of 5 mM Tris-HCl. Proteolytic digestion was performed overnight at 37 °C by adding 100 µL of a combined solution of endoproteinase Lys-C (Wako^®^, Richmond, VA, USA) and mass spectrometry grade trypsin (Pierce™, ThermoFisher Scientific, USA) in an enzyme/protein ratio of 1:50 *w/w* on top of the filters. The resulting peptides were recovered by centrifugation. The devices were then rinsed with 50 µL of 4% trifluoroacetic acid (ThermoFisher Scientific, USA) and centrifuged. This step was repeated three times, the peptides were finally desalted on SDB-RPS StageTips (Empore™, 3M^®^, Saint Paul, MN, USA), and were then dried by vacuum centrifugation [50].

For TMT labelling, dried peptides were first reconstituted in 10 μL of 100 mM Hepes buffer (Sigma-Aldrich^®^, USA) at pH 8, and 4 μL of TMT (ThermoFisher Scientific, USA) solution (i.e., 25 µg/μL in pure acetonitrile, Biosolve^®^, Dieuze, France) were then added. TMT labelling was performed at ambient temperature for 90 min and reactions were quenched with hydroxylamine (ThermoFisher Scientific, USA) to a final concentration of 0.4% *v/v* for 15 min. TMT-labeled samples were then pooled at a 1:1 ratio across all samples and desalted on SDB-RPS StageTips (3M^®^, USA). A single shot control LC-MS run was performed to ensure similar peptide mixing across each TMT channel and to avoid the need for further excessive normalization. Quantities of each TMT-labeled sample were adjusted according to the results of the control run. The combined samples were then desalted using a 100 mg SEP-PAK^®^ C18 cartridge (Waters, Milford, MA, USA) and were vacuum centrifuged. Pooled samples were fractionated into 12 fractions using an Agilent OFF-Gel 3100 system (Agilent, Santa Clara, CA, USA) following the manufacturer’s instructions. The resulting fractions were dried by vacuum centrifugation and desalted again on SDB-RPS StageTips (3M^®^, USA).

#### 2.6.2. LC-MS/MS Processing of Samples

Each individual fraction was resuspended in 2% acetonitrile and 0.1% formic acid (Merck-Millipore^®^, USA), and nano-flow separations were performed on a Dionex™ Ultimate 3000 RSLC nano UPLC system (ThermoFisher Scientific, USA) connected on-line with an Exploris™ 480 Orbitrap mass spectrometer (ThermoFisher Scientific, USA). A capillary precolumn (Acclaim™ PepMap™ C18; 3 μm-100 Å; 2 cm × 75 μm ID; ThermoFisher Scientific, USA) was used for sample trapping and cleaning. Analytical separations were performed at 250 nL/min over a 150 min biphasic gradient on a 50 cm long in-house packed capillary column (75 μm ID; ReproSil-Pur C18-AQ; 1.9 μm silica beads; Dr. Maisch, Ammerbuch, Germany). Acquisitions were performed through Top Speed Data-Dependent acquisition mode using a 3 s cycle time. Initial MS scans were acquired at a resolution of 120,000 (i.e., at 200 m/z) and the most intense parent ions were selected and fragmented by High-Energy Collision Dissociation (HCD) with a Normalized Collision Energy (NCE) of 36%, using an isolation window of 0.7 m/z. Fragmented ion scans were acquired with a resolution of 30,000 (i.e., at 200 m/z) enabling use of the turbo TMT mode, and selected ions were then excluded for the following 45 s. The corresponding mass spectrometry proteomics data were deposited at the ProteomeXchange Consortium (http://www.proteomexchange.org/, accessed 20 September 2021) via the PRIDE partner repository with the dataset identifier PXD028359.

#### 2.6.3. LC-MS/MS Data Analysis

Raw data were processed using SEQUEST, Mascot, MS Amanda, and MS Fragger in Proteome Discoverer™ version 2.4 (ThermoFisher Scientific, USA) against the human reference proteome database (i.e., 74,468 sequences) [51,52]. Enzyme specificity was set to trypsin and a minimum of six amino acids was required for peptide identification. Up to two missed cleavages were allowed, and a 1% false discovery rate (FDR) cut-off was applied both at peptide and protein identification levels. For the database search, carbamidomethylation (C), TMT tags (i.e., K- and peptide N-termini) were set as fixed modifications, whereas oxidation (M) was considered as a variable one.

Resulting text files were processed through in-house written R scripts (version 3.6.3, R Foundation for Statistical Computing, Vienna, Austria) [53]. A first normalization step was applied according to Sample Loading normalization [54]. Assuming that the total protein abundances were equal across the TMT channels, the reporter ion intensities of all spectra were summed and each channel was scaled according to this sum so that the sum of reporter ion signals per channel equaled the average of the signals across all samples. A Trimmed M-Mean normalization step was also applied using the EdgeR package (version 3.26.8) [55]. Assuming that samples contained a majority of non-differentially expressed proteins, this second step calculated normalization factors according to these presumed unchanged protein abundances. Proteins with high or low abundances and proteins with larger or smaller fold changes were not considered. Differential protein expression analysis was performed using the R bioconductor package limma (version 3.34.9, 22.02.2018), followed by the Benjamini–Hochberg multiple-testing method [56,57]. The GOATOOLS library (version 1.0.3) in Python was used for Gene Ontology enrichment analysis of the significant proteins (i.e., with an FDR < 0.01 and log_2_ fold changes (FC) greater than 0.9 in value or less than −0.9 in value) [58].

### 2.7. Specific ECM Protein Quantification in hFPT Lysates

#### 2.7.1. Collagen I Western Blotting

hFPTs were harvested from normoxic and hypoxic confluent T75 flasks (TPP, Switzerland) after 14 ± 2 days of culture, and lysed using RIPA lysis buffer (Abcam, UK) supplemented with 1 × protease inhibitors (Promega Corporation, USA). After an incubation period of 15 min on ice, the cell lysates were centrifuged at 10^3^× *g* for 5 min at ambient temperature. Supernatants were then transferred to new test tubes. The total protein contents in the samples were determined using a Pierce™ BCA Protein assay kit (ThermoFisher Scientific, USA), according to the manufacturer’s protocol and specifications. Quantities of 15 µg of total protein/sample were subsequently separated by electrophoresis on NuPAGE™ 4–12% Bis-tris polyacrylamid gels (ThermoFisher Scientific, USA), before being transferred onto nitrocellulose membranes (Cytiva, USA). The membranes were blocked with PBS-Tween^®^ 20 at 0.05% *v/v* (Applichem, Germany) supplemented with 5% *w/v* skim milk (Régilait, France) for 15 min at ambient temperature, and were incubated overnight at 4 °C with primary antibodies, namely anti-col1 (ref. ab34710, Abcam, UK) or anti-actin (ref. PA1-21167, ThermoFisher Scientific, USA). The membranes were then washed thrice with PBS-Tween^®^ 20 buffer and incubated for 1 h at ambient temperature with a secondary HRP-anti-rabbit antibody (ref. 20403, Biotium Inc., USA). The membranes were finally developed using the chemiluminescence detection system ECL™ Prime (Cytiva, USA), as previously described.

#### 2.7.2. hFPT Freeze-Thaw Lysate Preparation

hFPTs were harvested from confluent normoxic and hypoxic T75 flasks (TPP, Switzerland) after 14 ± 2 days of culture and washed with PBS (Bichsel, Switzerland) to remove serum traces. Cells were resuspended in PBS (Bichsel, Switzerland) at a concentration of 10^7^ cells/mL and submitted to freeze-thaw lysis by three successive transfers from liquid nitrogen to a waterbath set at 37 °C. The total protein contents in the samples were then determined using the Pierce™ BCA Protein assay (ThermoFisher Scientific, USA), as previously described.

#### 2.7.3. Comparative Endpoint Quantification of Elastin by Colorimetry

Elastin contents were respectively determined in hFPT normoxic and hypoxic freeze-thaw lysates using a Fastin™ Elastin assay (Biocolor Ltd., Carrickfergus, UK), according to the manufacturer’s protocol and specifications. Briefly, hFPT lysates were treated with oxalic acid for 1 h at 100 °C, before being centrifuged at 10^4^× *g* for 10 min at ambient temperature. Equal volumes of elastin precipitating reagent were then added to the isolated supernatants and the samples were incubated for 15 min. The samples were then centrifuged at 10^4^× *g* for 10 min before the supernatants were discarded. Residual materials were incubated for 90 min with 1 mL of dye reagent before being centrifuged at 10^4^× *g* for 10 min. Supernatants were discarded and the samples were then incubated with 250 µL of dye dissociation solution until the dye was completely released from the residual materials. The samples were then transferred in a 96-well microplate (Greiner, Frickenhausen, Germany) and absorbance measurements were performed at a wavelength of 513 nm on a Varioskan™ LUX multimode plate reader (ThermoFisher Scientific, USA). Data were analyzed using Skanit software (ThermoFisher Scientific, USA) with a linear regression curve. The elastin contents in each sample were normalized to the total protein contents.

#### 2.7.4. Comparative Endpoint Quantification of Fibronectin by ELISA

Fibronectin contents were respectively determined in hFPT normoxic and hypoxic freeze-thaw lysates using the DuoSet ELISA human fibronectin kit (R&D Systems, Minneapolis, MN, USA), according to the manufacturer’s protocol and specifications. For data acquisition, the absorbance measurements were performed at a wavelength of 450 nm on a Varioskan™ LUX multimode plate reader (ThermoFisher Scientific, USA) in 96-well ELISA microplates (Greiner, Germany). The data were analyzed using the Skanit software (ThermoFisher Scientific, USA) using a 4PL regression curve. The fibronectin contents in each sample were normalized to the total protein contents.

#### 2.7.5. Comparative Endpoint Quantification of Total GAGs by Colorimetry

Total GAG contents were respectively determined in hFPT normoxic and hypoxic freeze-thaw lysates using the Glycosaminoglycan Assay Blyscan™ (Biocolor, UK), according to the manufacturer’s protocol and specifications. Briefly, cell lysates were digested with a papain digestion solution for 3 h at 65 °C and then centrifuged at 1.2 × 10^4^× *g* for 10 min at ambient temperature. Then, volumes of 100 µL of supernatant were mixed with 1 mL of Blyscan™ dye reagent and samples were incubated for 30 min at ambient temperature. The samples were then centrifuged at 1.2 × 10^4^× *g* for 10 min at ambient temperature. Supernatants were discarded and 0.5 mL of dissociation reagent was added to the pellets. Samples were then incubated until the dye was completely released from the pellets. The samples were then transferred onto a 96-well microplate (Greiner, Germany) and absorbance values were measured at a wavelength of 656 nm on a Varioskan™ LUX multimode plate reader (ThermoFisher Scientific, USA). The data were analyzed using the Skanit software (ThermoFisher Scientific, USA) with a linear regression curve. The total GAG contents in each sample were normalized to the total protein contents.

### 2.8. Statistical Analysis

Experiments were performed in triplicate unless specified otherwise. For statistical comparison of the average values from two sets of data, a paired Student’s *t*-test was applied, following appropriate evaluation of the normal distribution of data, wherein a *p*-value < 0.05 was retained as a base for statistical significance determination. For statistical comparison of the values from multiple sets of quantitative data from experiments wherein multiple variables applied (e.g., multiple groups, various treatments), a one-way ANOVA test was performed and was followed (when appropriate) by a post hoc Tukey’s multiple comparison test, wherein a *p*-value < 0.05 was retained as a base for statistical significance determination. The calculations were performed using Excel (Microsoft Corporation, Redmond, WA, USA) and GraphPad Prism version 8.0.2 (GraphPad Software, Inc., San Diego, CA, USA).

## 3. Results

### 3.1. Hypoxia Transiently Induces HIF-1α Expression in hFPTs

**Figure 1:**
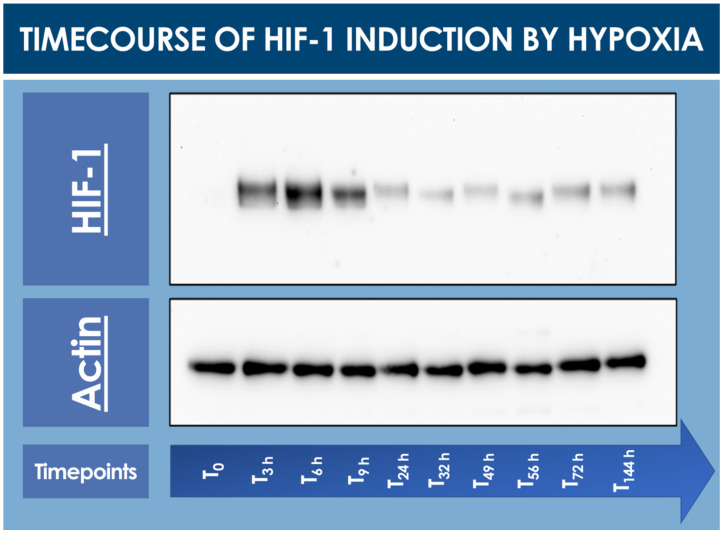
Timecourse specific analysis of HIF-1α induction upon hFPT (i.e., cells expanded to 80% confluency in normoxia; 5% CO_2_, 21% O_2_) exposure to hypoxia incubation conditions (i.e., 5% CO_2_, 2% O_2_). T_0_ represents the normoxic culture harvest initial timepoint (i.e., no hypoxic incubation phase). Subsequent timepoints represent hypoxic culture harvests. Whole gel imaging is provided in Supplementary Materials, Figure S1. Quantitative results of relative HIF-1 detection are presented in Supplementary Materials, Table S1. hFPT, human fetal progenitor tenocytes; HIF, hypoxia-inducible factor.

To confirm that the hFPTs responded to cell culture transition from normoxia to hypoxia, a timecourse assay of HIF-1α induction was performed and monitored by Western blot analysis. In normoxia (i.e., T_0_ timepoint), no HIF-1α was detected in the cell lysate, while a strong induction of the protein of interest was already observed after 3 h and up to 9 h in hypoxic conditions (Figure 1, Table S1).

Relatively elevated HIF-1α levels already started to decrease after 24 h of low O_2_ exposure and were thereafter consistent up to 144 h at the end of the timecourse (Figure 1).

### 3.2. hFPT Comparative Proliferation Assays and Cell Size Distribution Analysis

**Figure 2:**
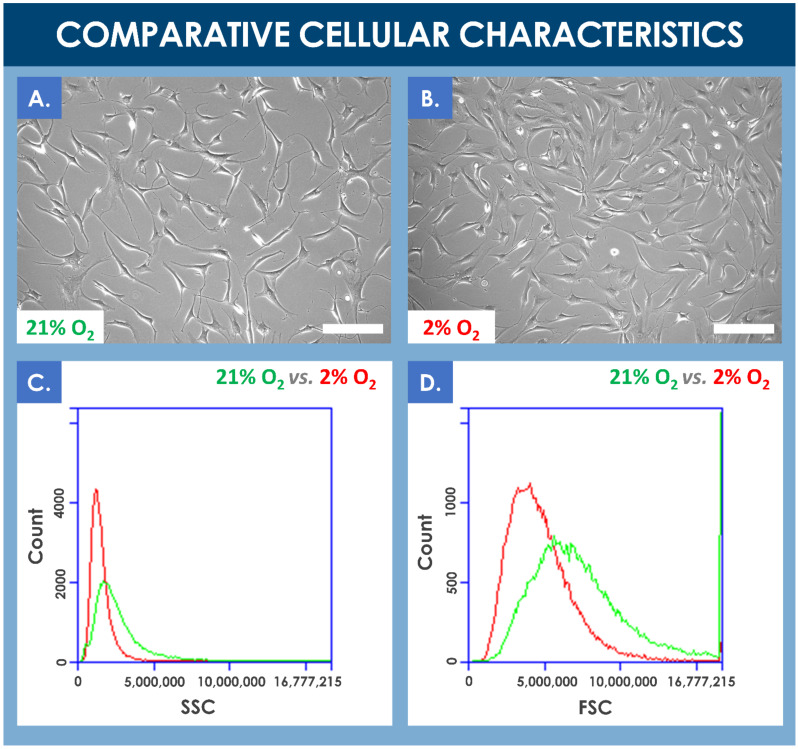
(**A**,**B**) Comparison of the proliferative cellular morphologies of hFPTs incubated in normoxia conditions (i.e., 5% CO_2_, 21% O_2_) or in hypoxia conditions (i.e., 5% CO_2_, 2% O_2_) for 6 days. Scale bars = 200 µm. (**C**) Comparative SSC plots of hFPTs cultured in hypoxia conditions (i.e., in red) and in normoxia conditions (i.e., in green). SSC or side scatter provides information about granularity and its distribution for the considered cells. Comparative analysis of both population profiles indicated that cell populations cultured in hypoxia were relatively less granular and more homogenous in their granularity distribution. (**D**) Comparative FSC plots of hFPTs cultured in hypoxia conditions (i.e., in red) and in normoxia conditions (i.e., in green). FSC or front scatter provides information about size and its distribution for the considered cells. Comparative analysis of both population profiles indicated that cell populations cultured in hypoxia were relatively smaller in size and more homogenous in their size distribution. The detected signal spike near the upper limit of the FSC channel may have been caused by the presence of large aggregates in the sample from the normoxia group. FSC, forward scatter; hFPT, human fetal progenitor tenocytes; SSC, side scatter.

Results of the comparative proliferation assays did not reveal any significant visual phenotypic differences between normoxic and hypoxic conditions wherein the proliferating hFPTs conserved their characteristic spindle-shaped and elongated morphology (Figure 2A,B, Process Parameter Supplementary Document). However, cell population profiles analyzed by flow cytometry revealed a relative data shift between the two culture conditions. In hypoxia conditions, the analyzed cell population was more homogenous, cell size was relatively smaller, and cell granularity was relatively reduced (Figure 2C,D). Furthermore, a comparative analysis of mean relative cell sizes between incubation conditions revealed a statistically significant inferior mean cell size for the hypoxia group (i.e., mean cell size of 157 ± 49 relative units) as compared to the normoxia group (i.e., mean cell size of 134 ± 29 relative units, determined from five photographs from each group, with 10 measurements per photograph).

**Figure 3:**
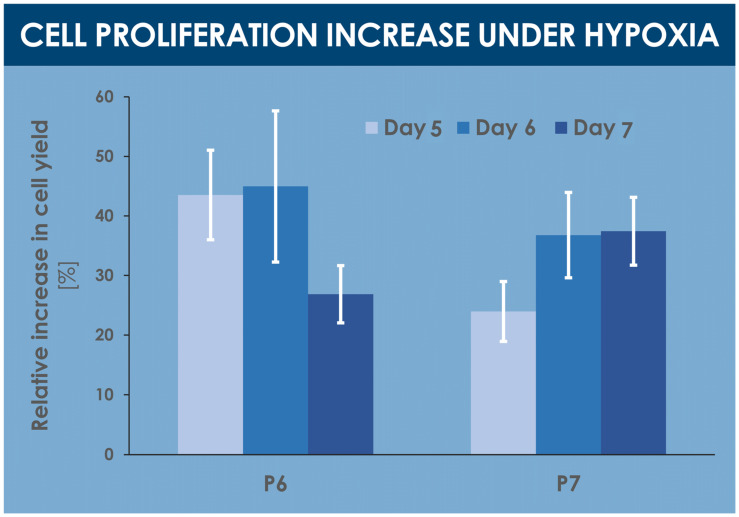
Comparative results of proliferative and endpoint cell yields, presented as the mean relative cell yield increases observed in hypoxia conditions vs. normoxia conditions. Various harvest endpoints were investigated for hFPTs at passage levels of 6 and 7, respectively. Results are presented as mean values, with corresponding standard deviations as error bars. hFPT, human fetal progenitor tenocytes; P, passage level.

A comparative analysis of various proliferative and endpoint cell yields indicated significant relative yield increases under hypoxic conditions, between 25% and 45%, for various specified harvest timepoints within the culture period (i.e., days 5, 6, and 7 of culture) and cell passage levels (i.e., passages 6 and 7, Figure 3, Table 1). Similarly, relatively higher cell yields were obtained in confluent hypoxic T75 flasks as compared to normoxic T75 flasks (data not shown).

### 3.3. hFPT Comparative Surface Marker Panel Characterization by Flow Cytometry

**Figure 4:**
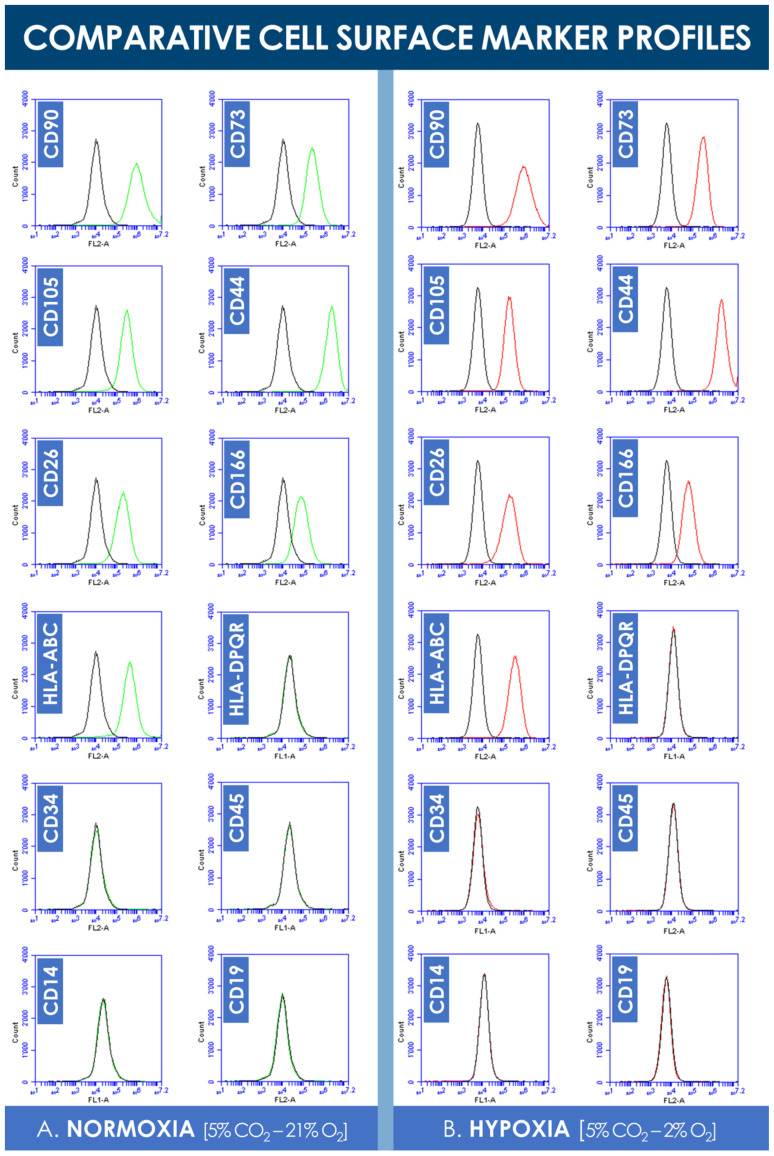
Comparative hFPT surface maker panel expression in normoxic and hypoxic culture conditions. (**A**) Representative flow cytometry data of cell surface marker expression of hFPTs cultured in a normoxic gaseous mix (i.e., green plots) as compared to isotype controls (i.e., black plots). (**B**) Representative flow cytometry data of cell surface marker expression of hFPTs cultured in a hypoxic gaseous mix (i.e., red plots) as compared to isotype controls (i.e., black plots). hFPT, human fetal progenitor tenocytes.

Analysis of the flow cytometry data did not reveal significant shifts in cell surface marker panel expression when comparing normoxia and hypoxia incubation conditions (Figure 4). Consistent with existing characterization data, hFPTs positively expressed clusters of differentiation CD90, CD73, CD105, HLA-ABC, CD26, CD166, and CD44 in both culture conditions (Figure 4). Both sample groups were found to not express clusters of differentiation CD19, CD14, CD34, CD45, and HLA-DPQR, which reflect immune system or vascular-related cell typologies (Figure 4).

### 3.4. hFPT Comparative Phenotypic Stability in Chemical Adipogenesis and Osteogenesis Induction Models

**Figure 5:**
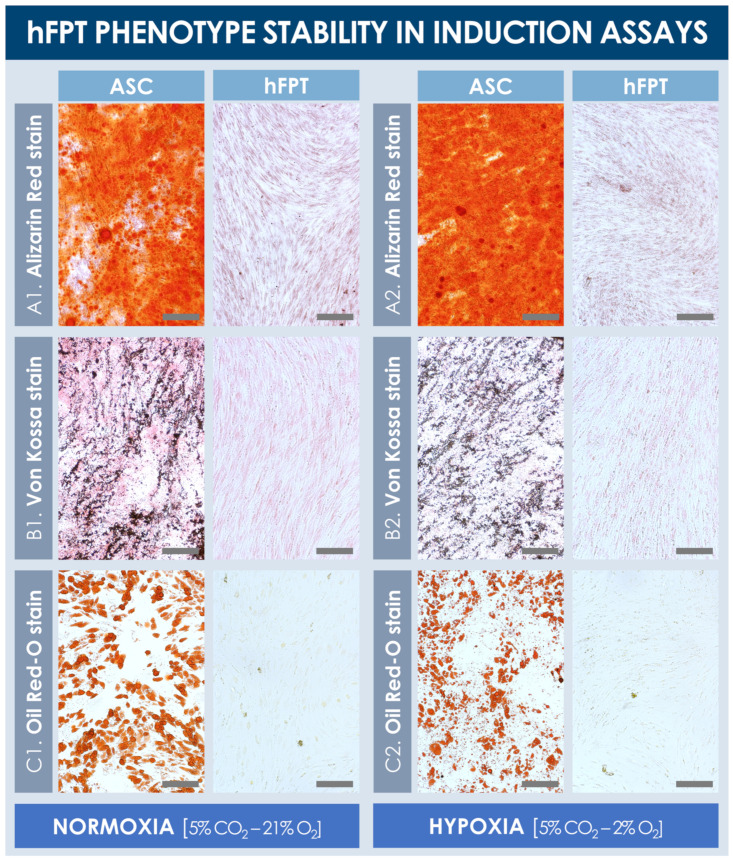
Comparative hFPT phenotype stability in chemical osteogenic and adipogenic induction assays. Representative imaging is provided for ASCs (i.e., positive controls) and hFPTs induced in osteogenic medium under both normoxia and hypoxia conditions, respectively, with Alizarin Red staining (**A**) or Von Kossa staining (**B**) of mineralized matrix, respectively. (**C**) Representative imaging of ASCs (i.e., positive controls) and hFPTs induced in adipogenic medium under both normoxia and hypoxia conditions, respectively, with Oil Red-O staining of lipid droplets. Scale bars = 200 µm. ASC, adipose-derived stem cells; hFPT, human fetal progenitor tenocytes.

Results from the chemical induction assays from both sample groups revealed that hFPTs cultured in normoxia conditions or in hypoxia conditions were not capable of producing lipid droplets or a mineralized matrix (i.e., calcium and phosphate depositions) in adipogenic and osteogenic induction conditions, respectively (Figure 5). In contrast, ASC-F positive controls produced large quantities of a mineralized matrix in both normoxia and hypoxia culture conditions, as visualized following Alizarin Red and Von Kossa staining (Figure 5A,B). Similarly, ASCs proliferated well and produced large lipid droplets in both culture conditions (i.e., with relatively smaller and more diffuse droplet patterns in hypoxia conditions), as revealed by Oil Red-O staining (Figure 5C). Notably, hypoxic culture conditions did not adversely impact the in vitro expansion of ASCs’ positive controls, and no observable differences were noted between both respective positive control groups.

### 3.5. hFPT Comparative Proteomic Analysis by Quantitative Mass Spectrometry

**Figure 6:**
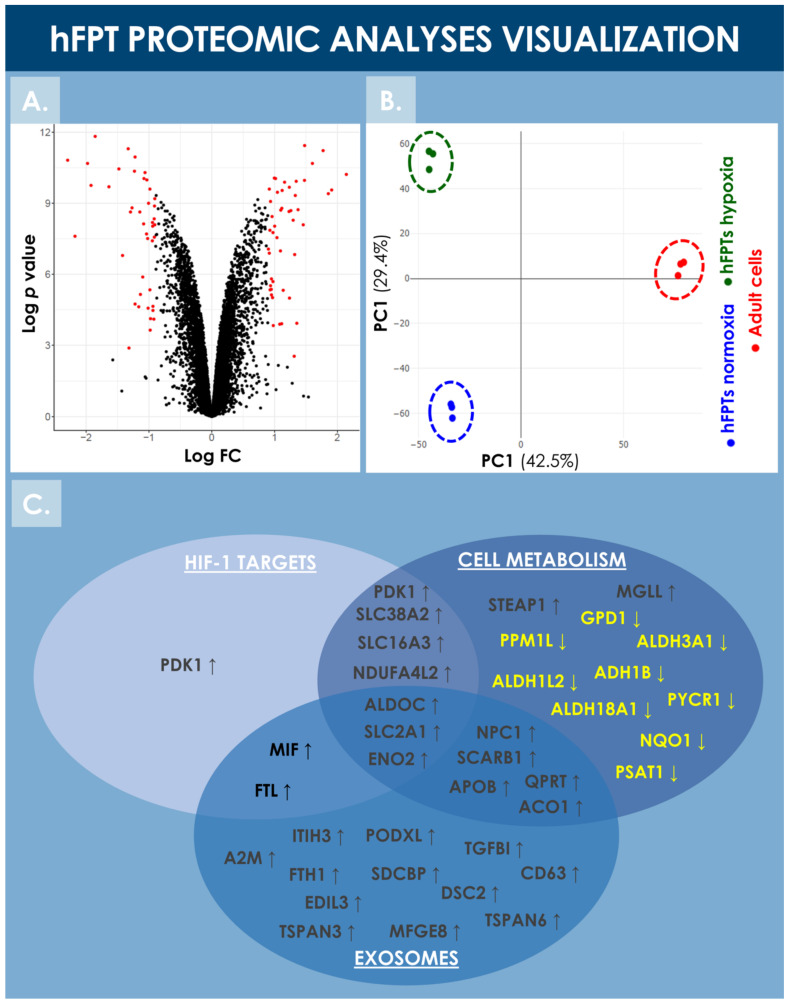
Visualization of the comparative proteomic analyses of hFPTs cultured in hypoxia conditions as compared to hFPTs cultured in normoxia conditions. (**A**) Volcano plot representing the log_2_ FC values (i.e., x-axis) assorted to log *p*-values (i.e., y-axis) of hFPT proteins from the hypoxia sample group as compared to the normoxia sample group. Data points related to protein hits with a significance threshold value specified at −0.9 for downregulation and at 0.9 for upregulation, and an FDR threshold value specified at ≤0.01, are presented in red. (**B**) Principal component analysis biplot representing scores of hFPTs cultured in hypoxia (i.e., green data points) and normoxia (i.e., blue data points) conditions, as well as the scores of adult tenocytes (i.e., red data points) cultured in normoxia and included as controls. (**C**) Venn diagram representing upregulated and downregulated proteins from the hFPT hypoxia sample group as compared to the normoxia hFPT sample group, with regard to respective implications in cellular metabolism mechanisms, exosome production, and hypoxia-responsive mechanisms (e.g., mediated by HIF-1α). FC, fold change; FDR, false discovery rate; hFPT, human fetal progenitor tenocytes; PC, principal component.

hFPTs cultured in normoxic and hypoxic conditions were analyzed by LC-MS/MS and their respective proteomes were compared in order to identify differentially regulated proteins. Proteomic analyses identified more than 8000 proteins in both hFPT culture conditions (Supplementary Materials, Spreadsheet S1). Following appropriate statistical validation, comparative quantitative data were expressed as a base 2 logarithm of the fold change (i.e., log_2_ FC) for the detected proteins. Threshold values of 0.9 or −0.9 were specified for the log_2_ FC and with an FDR ≤ 0.01 to define statistical significance with regard to differential protein expression. During the analysis, 47 upregulated proteins and 42 downregulated proteins were identified, as visualized by volcano plot representation (Figure 6A). The identified upregulated and downregulated proteins are listed in Table 2 and Table 3, respectively, along with their respective log_2_ FC ratio.

Notably, HIF-1α was not identified during the endpoint proteomic analysis in the hFPT cells cultured in hypoxia for two weeks (i.e., until reaching confluency), reflecting the transient and early activation of this master transcriptional switch during hypoxia [59]. However, well-known direct HIF-1α targets were identified in the upregulated protein list, such as PDK1, SLC2A1, SLC38A2, and NDUFA4L2, confirming that hFPTs adapted their physiology to the hypoxic environment (Figure 6C, Table 2) [39,42,60,61,62,63]. Proteins involved in cellular energy metabolism were found to be modulated in the hypoxia group, suggesting that hFPTs modified their metabolism to increase glycolysis over mitochondrial respiration, a strong oxygen consumer and reactive oxygen species (ROS) producer. Specifically, SLC2A1, ENO2, ALDOC (i.e., involved in glycolysis), PDK1 (i.e., an inhibitor of the TCA cycle), and SLC16A3 (i.e., involved in lactate efflux) were strongly upregulated in the hypoxia group (Figure 6C, Table 2). Similarly, NDUFA4L2, a known inhibitor of the electron transport chain (ETC) complex I, was upregulated, while NDUF4, a subunit of ETC complex IV, was downregulated in the hypoxia group (Figure 6C, Table 2 and Table 3).

Proteins involved in cell cycle regulation and cell proliferation were also found to be upregulated in hypoxia conditions, correlating with the increase in cell proliferation observed in hypoxic environments (Figure 3, Table 2). The proteins CLSPN, WHD1, AURKA, CEP55, and SKA2 are well-known to be involved in mitosis processes, and PDK1 not only plays a role in the regulation of cellular metabolism but is also involved in cell proliferation by controlling the expression of cyclin D1 and p27 [64,65,66,67,68,69]. Similarly, CEND8 downregulation could also promote cell proliferation, as observed in neuronal precursors where an inverse correlation between CEND8 expression and cell proliferation or terminal differentiation is documented [70].

**Figure 7:**
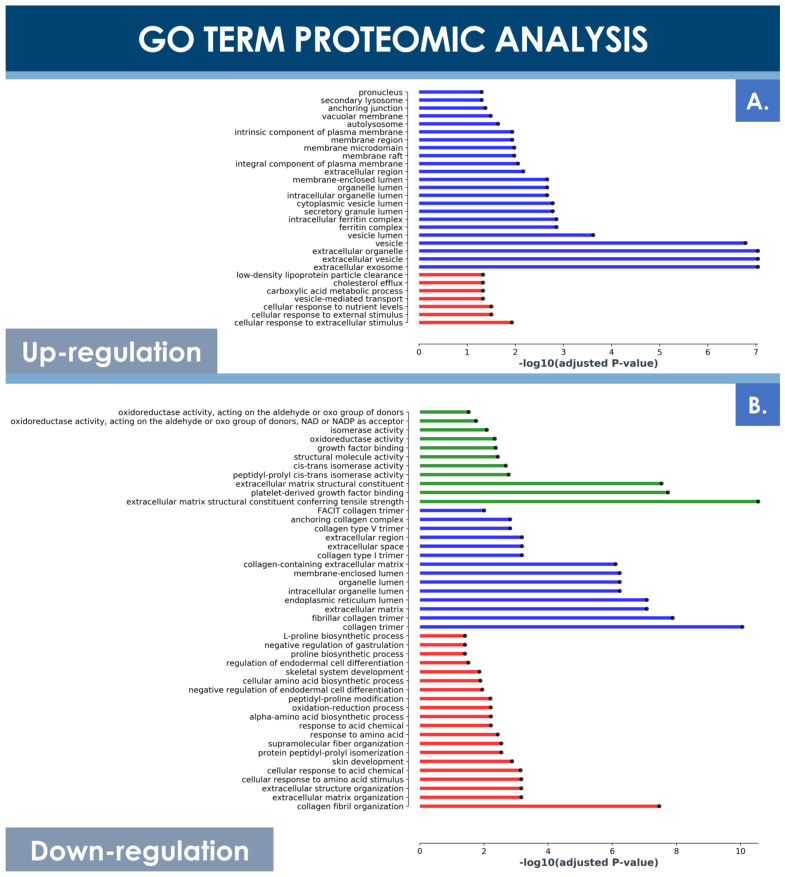
GO TERMS analysis of differentially expressed hFPT proteins in the hypoxia sample group as compared to the normoxia sample group, with a log significance threshold value specified at −0.9 for downregulation and at 0.9 for upregulation and an FDR significance threshold value specified at ≤0.01. (**A**) Upregulated protein GO TERMS. (**B**) Downregulated protein GO TERMS. Identified hits with relevant changes in detected levels were classified into three groups, namely “cellular compartment” (i.e., in blue), “molecular function” (i.e., in green), and “biological processes” (i.e., in red). FDR, false discovery rate; hFPT, human fetal progenitor tenocytes.

Principal component analysis (PCA) of both hFPT sample groups outlined clear differences with regard to protein expression levels between normoxic and hypoxic culture conditions, as well as clear differences between these two groups and an adult tenocyte sample (i.e., Ad-Ten-001 cells, cultured in normoxia) included as a control, respectively (Figure 6B). A specific GO TERMS enrichment analysis of the upregulated and downregulated proteins between hypoxic and normoxic hFPT groups was performed. An upregulated GO TERMS analysis highlighted the extracellular exosome compartment (Figure 7A) with proteins such as CD63, A2M, SDCBP, or MFGE8, which belong to the top 100 proteins identified in exosomes (ExoCarta database; http://www.exocarta.org/, accessed 3 August 2021), or APOB, NPC1, and SCARB1, involved in cholesterol trafficking (Figure 6C). A downregulated GO TERMS analysis evidenced proteins with oxidoreductase activity (i.e., ADH1B, NQO1, GPD1, ALDH3A1, ALDH18A1) (Figure 6C and Figure 7B), but the main effect was evidenced for collagen proteins, which were mostly downregulated in the hypoxia group (Table 4).

### 3.6. hFPT Specific ECM Protein Comparative Endpoint Analysis

**Figure 8:**
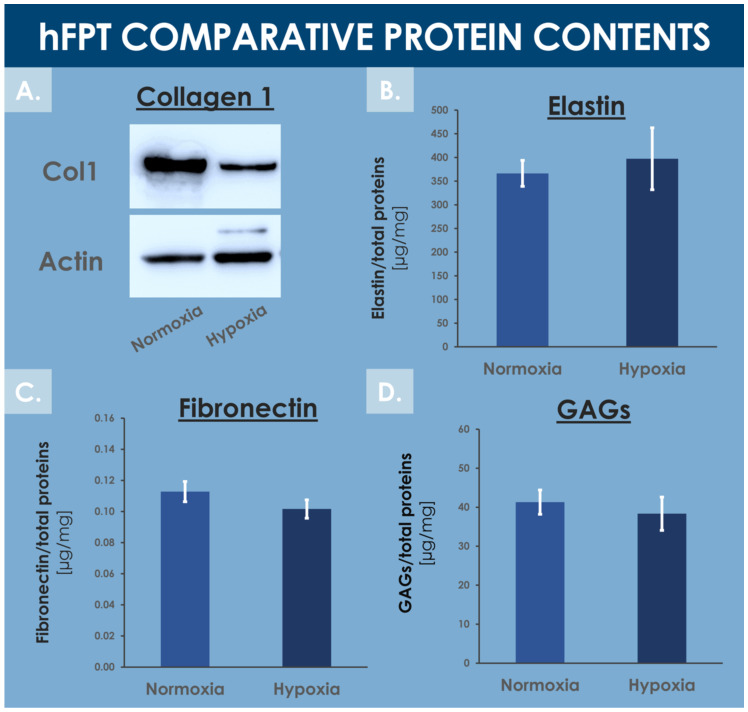
Comparative data relative to specific hFPT lysate protein contents in the normoxia incubation condition and in the hypoxia incubation condition. (**A**) Western blotting revealed a relatively lower collagen 1 content in the hypoxia sample group. Whole gel imaging can be found in Supplementary Materials, Figure S2. (**B**) Colorimetric measurements revealed conserved total elastin quantities in both sample groups (i.e., equality of variances and means, *p* = 0.157). (**C**) ELISA results revealed conserved total fibronectin quantities in both sample groups (i.e., equality of variances and means, *p* = 0.417). (**D**) Colorimetric measurements revealed conserved total GAG quantities in both sample groups (i.e., equality of variances and means, *p* = 0.304). ELISA, enzyme-linked immunosorbent assay; GAG, glycosaminoglycan; hFPT, human fetal progenitor tenocytes.

Tendons are mainly composed of water (i.e., 55–70%) and extracellular matrix proteins, with collagen 1 being the most abundant ECM protein [71,72]. The MS proteomic data along with complementary protein analysis allowed for direct ECM content comparison between experimental groups in order to evaluate the impact of hFPT culture conditions on their respective expression levels. Fibronectin quantification by ELISA and MS proteomic data (i.e., log_2_ FC value of −0.3) showed no statistical difference in fibronectin content between normoxia and hypoxia conditions (Supplementary Materials, Spreadsheet S1 and Figure 8C).

Colorimetric assays did not enable the detection of significantly different levels of total GAGs between both hFPT sample groups (Figure 8D). More specifically, the MS proteomic data comparison of specific proteoglycan, such as lumican, versican, biglycan, and decorin, did not show statistically significant modifications in protein expression between sample groups, with log_2_ FC values of −0.27, 0.18, 0.06, and 0.36, respectively (Supplementary Materials, Spreadsheet S1). Elastin was not identified in the MS datasets, probably due to its resistance to trypsin digestion. Elastin levels quantified by colorimetric assays in hFPT lysates did not show any impact of hypoxia on elastin expression as compared to the normoxia sample group (Figure 8B).

In contrast, the MS proteomic GO TERMS data analysis of hits with a significance threshold value specified at ≥0.9 for upregulation or ≤−0.9 for downregulation demonstrated a strong negative impact of hypoxia on collagen expression. Indeed, most of the downregulated GO TERMS (i.e., collagen fibril organization, platelet-derived growth factor binding, extracellular matrix structural constituents) were associated with collagen subtypes (Figure 7B). Direct log_2_ FC comparison of all collagen types identified in both hFPT proteome datasets showed a strong downregulation of COL1, COL3, COL4, COL5, COL12, and COL14 (Table 4). This observation was also confirmed for collagen 1 by Western blot analysis in hFPT lysates (Figure 8A).

## 4. Discussion

### 4.1. Conservation of hFPT Critical Quality Attributes in Hypoxic Culture Conditions as Compared to Normoxia Culture Conditions

The results of the various comparative experiments presented in this study demonstrated that hypoxia in vitro culture conditions did not adversely impact selected critical quality attributes (i.e., specific morphology, adherent proliferative behavior, phenotypic stability toward chemical induction, cell surface marker profile, ECM protein contents) of hFPTs as compared to classical normoxia culture conditions (Figure 2, Figure 4, Figure 5, and Figure 8, Process Parameter Supplementary Document). Specifically, the results confirmed that the cells of interest did not suffer notable changes with regard to identity (i.e., cell surface markers and morphology) or potency (i.e., susceptibility toward adipogenic or osteogenic chemical induction) in hypoxic culture, which would have been an exclusion criterion from a quality and manufacturing process standpoint (Figure 2, Figure 4, and Figure 5). Therefore, based on said results, the switch from normoxic (i.e., 5% CO_2_; 21% O_2_) culture conditions to hypoxic (i.e., 5% CO_2_; 2% O_2_) culture conditions for hFPT manufacture may be technically considered, as it is not preliminarily ruled out for safety or quality reasons. However, further comparative characterization studies will be necessary in view of validating a change in the manufacturing process and following appropriate risk-based qualification (i.e., toxicity and tumorigenicity assays, effects on the total cellular in vitro lifespan at high passages).

The interest and technical advantages of applying pre-natal tissue-derived progenitors such as hFPTs to homologous therapeutic settings derive from their intrinsic tissue-specific (i.e., pre-terminally differentiated) phenotype, pre-immunocompetent status (i.e., generally observed for pre-natal tissues and cells), and high robustness in simple standard in vitro culture conditions (i.e., no need for defined media with specific growth factors or synthetic cocktails) [1,73]. Therein, allogenic homologous therapeutic approaches and products may be devised with highly limited risks of tumor formation or eliciting immune responses. Notably, hFPTs manufactured in normoxia conditions were recently documented as preliminarily safe for implantation in a rabbit patellar tendon defect model, wherein hyaluronic acid served as a functional delivery scaffold [5]. The proof of concept of progenitor tenocyte-based APIs for homologous allogenic treatment of tendon defects or affections has been established, albeit with a remaining margin of improvement for the technical optimization of the manufacturing process. Indeed, when considering the various steps of multi-tiered hFPT banking and in vitro culture expansions in particular, greatest attention must be paid to the retained technical specifications and materials used for said substantial manipulations.

In accordance with the proliferative behavior of comparable primary cell types, hFPTs may largely benefit from the stringent optimization of manufacturing technical specifications and the benchmarking of materials and consumables for the assurance of maximal safety and quality of the obtained cellular APIs. Therefore, along with the culture medium supplement (i.e., FBS reference and lot number), the gaseous mix composition of the inlet to production incubators was determined as key in obtaining high-quality and homogenous cell populations. The latter aspect was experimentally confirmed in the present study, with the comparative evaluation of 21% O_2_ vs. 2% O_2_ incubation conditions.

### 4.2. Optimization of hFPT Key Quality Attributes in Hypoxic Culture Conditions as Compared to Normoxia Culture Conditions

The results of the various experiments presented in this study demonstrated that hypoxia in vitro culture conditions exerted quantifiable and significant impacts on selected key quality attributes (i.e., production yields, cell size distribution and cell body homogeneity, production of sub-cellular components) of hFPTs as compared to classical normoxia in vitro culture conditions (Figure 2, Figure 3, Figure 6, and Figure 7, Process Parameter Supplementary Document). The identified relative modifications, such as increases in cell population homogeneity and production yields, may be interpreted as twofold, pertaining to the ease of demonstration of conserved quality assurance levels as well as the cost-effectiveness of API manufacture. Indeed, clean-room operation fees are major drivers of the direct production costs of the API in cell-based therapeutic product manufacture. Furthermore, preparation for the regulatory evaluation of established workflows and processes for API and finished product manufacturing are at the heart of development concerns for industries developing cell therapy products. Manufacturers bear the responsibility of defining their process parameters (i.e., driven by quality optimization and risk-minimization), the related target and limit values or specifications, and the acceptance criteria for the assurance of maximal and reproducible quality of production cell batches. Therefore, the implementation of differential parameters (e.g., incubation atmosphere gaseous composition) procuring tangible benefits in both the quality assurance and manufacturing efficiency domains should be subjected to extensive scrutiny. The example of 2% O_2_ hFPT in vitro incubation presented in this study helps to set forward a relatively enhanced cellular API homogeneity in a manufacturing setting relatively closer to the physiological conditions of normal tenocyte growth (i.e., relative hypoxia as compared to atmospheric O_2_ levels, between 1% and 5% O_2_ depending on the tendon tissue zone or structure and activity-related needs), and with a significantly enhanced production efficiency, with a relative increase of 25–45% in harvest cell yields at defined timepoints of the cell expansion phase in selected conditions (Figure 3) [74,75]. When considering endpoint cell harvest (i.e., upon reaching 90–100% monolayer confluency), the main technical advantage procured by hypoxic hFPT incubation was the reduction in the overall manufacturing period (i.e., cell confluency could be reached sooner), which may be translated in diminished infrastructure-related fixed costs. Additionally, with the relatively smaller cell size in hypoxic conditions versus normoxic conditions, the relative endpoint hFPT cell yield was comparatively greater after the same incubation time due to the availability of greater relative culture surfaces (Table 1, Figure 2 and Figure 3).

In addition to hFPT quality attributes related to cell population proliferative behavior or qualitative composition, functional quality attributes must be further taken into account as they represent the ultimate and clinically relevant endpoints enabling a judicious comparative assessment of different conditions for manufacturing process modification. As for comparable cell therapy approaches for tendon regeneration or repair promotion, the exact mechanism of action and the fate of exogenous hFPT therapeutic biological materials remain incompletely characterized [5]. The main putative effects of implanted cells consist of the generation and deposition of ECM in tendon fibers, naturally evolutive in nature and modality during the successive healing phases, and the paracrine modulation or mediation of the patient’s own healing mechanisms via balanced arrays of growth factors and cytokines or sub-cellular vesicles such as exosomes [1,5]. Further precise and pondered determination of the relative importance of such mechanisms (i.e., ECM deposition vs. therapeutic signal emission or modulation) of action of hFPTs should help to determine if hypoxic culture conditions procure tangible benefits in terms of API and finished product activity. Indeed, while the comparative assessment of the relative quantities of key proteins or protein types (e.g., GAGs, fibronectin, elastin) has not revealed significant differences between both sample groups in the present study, specific proteomic data tended to outline relative downregulation of several collagen types (Figure 8A, Table 4).

In the case of tendon ECM, synthesis and alignment of collagens play central roles in proper structure formation and function of fibers and whole tissular structures. In this context, the relative downregulation of hFPT collagen synthesis due to differential manufacturing conditions (e.g., hypoxia) might be considered as a negatively impacting functional outcome, yet the exact relationship between therapeutic cell ECM generation and endpoint tissular functionality is not well characterized for tendons. Indeed, such tissues undergo multiple phases of tissue repair and reorganization, and the exogenous supplementation of specific ECM components by therapeutic cells is in all probability of lesser importance than in the domain of cartilage tissue repair, wherein structural resistance and functional restoration are highly dependent on ECM formation [48]. In the case where the presence of ECM in relatively abundant quantities is necessary for the optimal function of progenitor tenocyte-based therapeutic products, specific attention will need to be paid to the optimal harvest timepoint during or following cell expansion. Indeed, during the in vitro proliferative phase of cell expansion, the generation of ECM is relatively less important than when the cells reach confluency, at which point they shift their main activities from proliferation to gain of function (i.e., ECM synthesis in the case of tenocytes).

As compared to cell therapies designed for systemic administration (e.g., intravenous infusions), application of therapeutic cells (e.g., PRP, stem cells) in tendon repair strategies require smaller overall doses as they are administrated locally [3]. While cell doses for systemic infusion may reach 2–3 × 10^7^ cells/kg of body weight in selected applications, tendon regenerative medicine workflows comprise unitary product doses generally inferior to 10^7^ cells [1,3,76]. Local administration is furthermore necessary when considering tendon tissues for simple pharmacokinetic reasons (i.e., poor cell distribution due to restricted tissue vascularization). Therefore, as such cell doses for local tendon treatment are relatively smaller, reported clinical outcomes would suggest that, rather than exerting beneficial effects by direct ECM deposition, such materials act by direct or indirect modulation of the local host/patient environment [5]. As mentioned previously, the effectors of such putative modulation or repair chaperonings are, in all probability, complex, acting in synergy and in interplay with local endogenous mediators and effectors of repair, relatively scarce in quasi-avascular tendon tissues. Therein, the effects of small signaling factors such as cytokines or sub-cellular vesicles such as exosomes may be considered as key in directing optimal tissue repair and restoration. By extrapolation, and to be harnessed in an acellular product development setting, such types of cell-derived, cell-free approaches (e.g., MSC-derived exosomes) have recently been thoroughly investigated in the context of post-COVID-19 respiratory syndrome management and the functional restoration promotion of impacted lung tissues [77]. Due to the apparent upregulation of exosome-related markers in hypoxia-cultured hFPTs, a similar approach may potentially be investigated for tenocyte-derived acellular API production, with postulated processing and/or regulatory advantages (Figure 6C and Figure 7A). Further characterization of such putative mechanisms of action of hFPTs should additionally enable the comparative evaluation of manufacturing culture conditions with regard to endpoint functionality. Specifically, such data will help to shed some light on the practical implications of downregulated collagens in hypoxia-cultured hFPTs, for example, which may or may not be of critical or key importance for eventual preclinical and clinical applications.

### 4.3. hFPT HIF-1α Pathway Transient Induction and Collagen Downregulation in Hypoxia Culture Conditions

Specific focus on the transient upregulation of HIF-1α in hFPTs transferred to hypoxic incubation conditions confirms its role as an early transcriptional switch that initiates the hFPT cellular response to hypoxia (Figure 1). While HIF-1 was not identified in the endpoint MS proteomic analysis presented in this study, well-known direct HIF-1 target proteins involved, amongst others, in cellular energy metabolism or cell proliferation were identified (Figure 6C). The fact that HIF-1 and many of the known HIF-1 targets were not identified in the endpoint MS proteomic analysis further supports the theory that HIF-1 plays a crucial yet indirect role in the observed modifications in endpoint protein expression (Figure 6C). Therein, such modifications may be the ultimate result of the initial HIF-1 upregulation, but the quantitative results determined in endpoint may be caused by or result from a number of intermediary steps within specific cellular process pathways.

It has been extensively documented that cells respond to reduced O_2_ availability by adapting their metabolism. Such responses are mainly initiated by the transcription factor HIF-1, which induces hypoxia-responsive target genes such as *Glut1*, *PDK1,* or *VEGF*. HIF-1 is a heterodimer constituting of two subunits, HIF-1α and HIF-1β. During normoxia cell expansion, HIF-1α is constantly targeted toward proteosomal degradation, while it is stabilized during hypoxia cell culture, allowing a rapid induction of HIF-1 targets. In this study, it could be demonstrated that hFPT exposure to 2% O_2_ conditions results in the activation of HIF-1-dependent pathways. HIF-1α stabilization was readily and transiently observed in the progenitor tenocytes upon exposure to low O_2_ conditions (Figure 1). HIF-1α stabilization was the strongest between the 3 h to the 9 h timepoints, but already declined at the 24 h timepoint, and the protein was not detected by MS proteomic analysis after 14 days in hypoxic culture. This result is in accordance with work from Uchida et al., who showed a reduction in HIF-1α mRNA and protein levels during prolonged low O_2_ exposure [59].

The MS proteomic data gathered in this study tend to highlight conserved quantitative compositions with regard to most ECM components between both sample groups except for collagen subtypes COL1, COL3, COL4, COL5, COL12, and COL14, which presented strong respective expression reductions in hypoxia conditions (Figure 8A and Table 4). Such collagens mainly play structural roles, as they constitute the ECM and may exert modulating effects on other collagen types in tendons and adjacent tissues. In particular, such findings about collagens involved in structural functions and ECM architecture were linked to the GO TERMS proteomic analysis results presented in Figure 7, which also indicated significant and specific downregulation of structural proteins and components. Therefore, it could be further interpreted that under the influence of hypoxia, hFPTs responded by shifting their activity from mainly being structural effectors, to activities mainly centered on cell metabolism. Combined with the fact that hypoxic culture conditions favored the proliferation of hFPTs to a considerable extent, it would indeed appear that said conditions specifically favor metabolic activities and processes of cell replication over those of function or ECM production (Figure 3). This aspect was substantiated by comparative MS proteomic data outlining the upregulation in hypoxic hFPT culture conditions of alternative pathways and markers linked to structural cellular components or metabolic responses active during proliferation (Table 2, Figure 6C and Figure 7A). Furthermore, MS proteomic data gathered for the hypoxia group pointed toward a downregulation of structural protein pathways and markers related to hFPT extra-cellular processes and interactions with ECM components (Table 3, Figure 7B).

Data from the principal component analysis confirmed several aspects regarding hFPTs in various culture conditions as compared to adult tenocytes cultured in normoxia (Figure 6B). Firstly, the results outlined a clear difference between hFPTs and adult tenocytes both grown in normoxia using the same consumables and technical specifications. Secondly, a clear difference was also observed between hFPTs cultured in normoxia vs. in hypoxia (Figure 6B). This set of data and its representation in a biplot helped to visually confirm significant differences between sample groups submitted for analysis, which may be explained by intrinsic differences between the cell types as well as induced differences depending on the manufacturing conditions. When put into perspective with the other results of the present study, such considerations confirmed that the modifications incurred by hypoxic culture on hFPTs, as compared to normoxic culture, were specific in nature and were significant in terms of amplitude (e.g., log_2_ FC values in Table 2, Table 3 and Table 4).

### 4.4. Specificities of hFPT Hypoxic Culture Conditions for Potential Next-Generation Manufacture of Cell-Free APIs in Tendon Regenerative Medicine

Based on the conserved critical quality attributes of hFPTs and relatively increased manufacturing yields in hypoxic culture conditions, further attention should be paid to the development of tenocyte-derived, cell-free APIs for tendon regenerative medicine. Although integral and viable cells are in all probability required for tissue engineering approaches in the case of volumetric tissue defects or large tendon ruptures, specific homologous and cell-free approaches may potentially be more suited for the management of subcritical defects, local inflammation, or as adjuvant therapies for the surgical management of tendons. Specifically, with regard to the comparative quantitative MS proteomic data gathered in this study, the potent induction of exosome-related markers and pathways would tend to orient further hFPT manufacturing optimization and characterization work toward injectable formulations exempt of whole-cell units (Figure 6 and Figure 7). Despite the additional complexity entailed by isolation and purification workflows for obtention of hypoxic hFPT exosomes, for example, such approaches may yield tangible benefits with regard to the quality assurance levels of finished products (i.e., possibly terminally filter-sterilized). Furthermore, the use of cell-based or cell-derived acellular APIs may contribute to alleviating some aspects of the regulatory process complexity classically put forward in standard cytotherapy approaches.

Overall, further technical optimization and in vitro characterization work is required with regard to hFPT culture parameter modification or the use of selected cell derivatives instead of whole viable cells. However, these developments and potential novel solutions in the field of tendon regenerative medicine need to be driven by tangible, functional endpoints and results, in addition to technical advantages or regulatory process simplifications. Indeed, time and costs may be critical parameters favoring or not favoring a specific developmental approach for eventual inclusion in translational settings, yet maximized product safety and quality remain as cornerstones for sound development of a clinically successful therapeutic product or therapy.

## 5. Conclusions

Cultured hFPTs have been established as potentially viable therapeutic candidates in the field of tendon regenerative medicine. Technical optimization of cell manufacturing processes may be further conducted in view of procuring additional benefits (i.e., technical, process-related, and functionally or clinically relevant). In particular, several complementary experimental methods and endpoints enabled the comparative assessment of the effects of hypoxic culture conditions on a progenitor tenocyte source of interest in this study. Stability of critical quality attributes and specific modifications of selected key quality attributes of FE002-Ten cells were demonstrated and discussed in 2% O_2_ vs. 21% O_2_ incubation conditions. Overall, this study indicated that hypoxic incubation tangibly and significantly impacts and modulates specific hFPT key properties and enables potential technical optimization for the manufacture of tenocyte-based APIs for homologous standardized transplant products. Based on detailed quantitative proteomic analyses and data, alternative API development processes or formulations may be considered in view of shifting from classical cytotherapy toward the development of cell-based, cell-free regenerative medicine solutions. Altogether, such potential therapeutic options may be of interest for the provision of complementary and putative holistic management solutions for tendon tissue wounds or affections.

## Figures and Tables

**Table 1:** Population doubling values for hFPTs harvested between days 5 and 7 of incubation in normoxic or hypoxic conditions at passage levels of 6 and 7, respectively. Statistically significant differences between mean values, determined using a unilateral paired *t*-test, are evidenced with an asterisk “*”. hFPT, human fetal progenitor tenocytes; SD, standard deviation.

		Population Doubling Values (Mean ± SD)			
		Passage 6 Cells	*p*-Values	Passage 7 Cells	*p*-Values
Day 5 harvest	21% O_2_	3.52 ± 0.22	0.012 *	2.98 ± 0.24	0.092
	2% O_2_	4.05 ± 0.12		3.30 ± 0.15	
Day 6 harvest	21% O_2_	3.83 ± 0.32	0.020 *	3.85 ± 0.12	0.165
	2% O_2_	4.37 ± 0.27		4.08 ± 0.37	
Day 7 harvest	21% O_2_	4.24 ± 0.23	0.016 *	4.29 ± 0.12	0.015 *
	2% O_2_	4.59 ± 0.10		4.74 ± 0.19	

**Table 2:** Data on relative protein expression upregulation in hFPTs cultured in hypoxia conditions as compared to hFPTs cultured in normoxia conditions. Relative quantitative data were expressed as fold change logarithms (i.e., base 2 log), with a significance threshold value specified at 0.9 for upregulation and an FDR threshold value specified at ≤0.01. Data were ranked according to their respective log_2_ FC values. Full datasets are available in Supplementary Materials, Spreadsheet S1. hFPTs, human fetal progenitor tenocytes; FC, fold change; FDR, false discovery rate.

Protein Name	Accession Number	Protein Symbol	Log_2_ FC Hypoxia vs. Normoxia
NADH dehydrogenase [ubiquinone] 1 alpha subcomplex subunit 4-like 2	Q9NRX3	NDUFA4L2	2.1464
Syndecan binding protein (Syntenin), isoform CRA_a	G5EA09	SDCBP	1.9118
Alpha-2-macroglobulin	P01023	A2M	1.8585
Ferritin heavy chain	P02794	FTH1	1.7748
Ferritin light chain	P02792	FTL	1.6041
Nicotinate-nucleotide pyrophosphorylase [carboxylating]	Q15274	QPRT	1.4797
Monocarboxylate transporter 4	O15427	SLC16A3	1.4795
Lysosomal-associated transmembrane protein 4A	Q15012	LAPTM4A	1.4593
Solute carrier family 2, facilitated glucose transporter member 1	P11166	SLC2A1	1.3763
Spindle and kinetochore-associated protein 2	J3KSP0	SKA2	1.3565
Transforming growth factor-beta-induced protein ig-h3	Q15582	TGFBI	1.3464
Lactadherin	Q08431	MFGE8	1.3313
Zinc finger protein 185	O15231	ZNF185	1.3219
Mitogen-activated protein kinase 13	O15264	MAPK13	1.3148
[Pyruvate dehydrogenase (acetyl-transferring)] kinase isozyme 1, mitochondrial	Q15118	PDK1	1.2980
Apolipoprotein B-100	P04114	APOB	1.2616
Metalloreductase STEAP1	Q9UHE8	STEAP1	1.2398
Tetraspanin (fragment)	F8VWK8	CD63	1.2375
Fos-related antigen 1	P15407	FOSL1	1.2331
Gamma-enolase	P09104	ENO2	1.1619
G-protein coupled receptor 39	O43194	GPR39	1.1402
Cytoplasmic aconitate hydratase	P21399	ACO1	1.1232
Podocalyxin	O00592	PODXL	1.1188
RNA-binding protein EWS (fragment)	H7BY36	EWSR1	1.1169
NPC intracellular cholesterol transporter 1	O15118	NPC1	1.1023
Tetraspanin-3	O60637	TSPAN3	1.0965
Repetin	Q6XPR3	RPTN	1.0841
EGF-like repeat and discoidin I-like domain-containing protein 3	O43854	EDIL3	1.0480
Sodium-coupled neutral amino acid transporter 2	Q96QD8	SLC38A2	1.0393
Fructose-bisphosphate aldolase C	P09972	ALDOC	1.0192
Desmocollin-2	A0A3B3ISU0	DSC2	1.0026
Inter-alpha-trypsin inhibitor heavy chain H3	A0A087WW43	ITIH3	0.9987
Hepatocyte growth factor activator	D6RAR4	HGFAC	0.9831
Centrosomal protein of 55 kDa	Q53EZ4	CEP55	0.9777
Claspin	Q9HAW4	CLSPN	0.9771
Macrophage migration inhibitory factor	P14174	MIF	0.9708
Rho GTPase-activating protein 7	Q96QB1	DLC1	0.9628
Aurora kinase A	O14965	AURKA	0.9528
Leucine-rich repeat and fibronectin type-III domain-containing protein 5	Q96NI6	LRFN5	0.9520
Proenkephalin-A	P01210	PENK	0.9503
Scavenger receptor class B member 1	B7ZKQ9	SCARB1/SR-B1	0.9385
Sortilin	Q99523	SORT1	0.9375
Tyrosine-protein kinase Fes/Fps	P07332	FES	0.9296
Tetraspanin-6	O43657	TSPAN6	0.9283
WD repeat and HMG-box DNA-binding protein 1	O75717	WDHD1	0.9185
Monoglyceride lipase	Q99685	MGLL	0.9183
Gamma-aminobutyric acid receptor-associated protein-like 2	P60520	GABARAPL2	0.9041

**Table 3:** Data on relative protein expression downregulation in hFPTs cultured in hypoxia conditions as compared to hFPTs cultured in normoxia conditions. Relative quantitative data were expressed as fold change logarithms (i.e., base 2 log) with a significance threshold value specified at −0.9 for downregulation and an FDR threshold value specified at ≤0.01. Data were ranked according to their respective log_2_ FC values. Full datasets are available in Supplementary Materials, Spreadsheet S1. hFPTs, human fetal progenitor tenocytes; FC, fold change; FDR, false discovery rate.

Protein Name	Accession Number	Protein Symbol	Log_2_ FC Hypoxia vs. Normoxia
All-trans-retinol dehydrogenase [NAD (+)] ADH1B	ADH1B	P00325	−2.2908
Ubiquilin-1 (fragment)	UBQLN1	H0YDS0	−2.1746
Collagen alpha-1(I) chain	COL1A1	P02452	−1.9743
Aldehyde dehydrogenase, dimeric NADP-preferring	ALDH3A1	P30838	−1.9191
Collagen alpha-1(XII) chain	COL12A1	Q99715	−1.8542
Limbic system-associated membrane protein	LSAMP	Q13449	−1.6349
Collagen alpha-2(I) chain	COL1A2	A0A087WTA8	−1.4767
Collagen alpha-1(XII) chain (fragment)	COL12A1	H0Y5N9	−1.4188
Collagen alpha-1(III) chain	COL3A1	P02461	−1.3271
Tropomodulin-1	TMOD1	P28289	−1.3166
Collagen triple helix repeat-containing protein 1	CTHRC1	Q96CG8	−1.2917
Collagen alpha-1(IV) chain	COL4A1	P02462	−1.2683
Isoform 2 of Collagen alpha-1 (V) chain	COL5A1	P20908-2	−1.2281
Neuroserpin	SERPINI1	Q99574	−1.2189
Transducin-like enhancer protein 4	TLE4	Q04727	−1.2179
BH3-interacting domain death agonist 1	BID	P55957	−1.1611
NAD(P)H dehydrogenase [quinone] 1	NQO1	P15559	−1.1425
Isoform TrkB-T1 of BDNF/NT-3 growth factors receptor	NTRK2	Q16620-2	−1.1343
Protein phosphatase 1L	PPM1L	Q5SGD2	−1.0958
Collagen alpha-2(V) chain	COL5A2	P05997	−1.0816
Collagen alpha-1(XIV) chain	COL14A1	Q05707	−1.0782
10-formyltetrahydrofolate dehydrogenase	ALDH1L2	A0A494C1M4	−1.0602
Cytochrome c oxidase subunit NDUFA4	NDUFA4	O00483	−1.0348
Peptidyl-prolyl cis-trans isomerase FKBP9	FKBP9	O95302	−1.0305
Fibroblast growth factor 1	FGF1	P05230	−1.0188
Cell cycle exit and neuronal differentiation protein 1	CEND1	Q8N111	−1.0105
Tissue alpha-L-fucosidase	FUCA1	P04066	−1.0054
Peptidyl-prolyl cis-trans isomerase FKBP10	FKBP10	Q96AY3	−0.9833
Alkaline phosphatase, tissue-nonspecific isozyme	ALPL	P05186	−0.9812
Glypican-4	GPC4	O75487	−0.9779
Peptidyl-prolyl cis-trans isomerase C	PPIC	P45877	−0.9732
Cystathionine beta-synthase-like protein	CBS	P0DN79	−0.9432
Protein disulfide-isomerase A5	PDIA5	Q14554	−0.9414
Pyrroline-5-carboxylate reductase 1, mitochondrial	PYCR1	P32322	−0.9383
Isoform 6 of Dystrophin	DMD	P11532-6	−0.9348
Glycerol-3-phosphate dehydrogenase [NAD (+)], cytoplasmic	GPD1	P21695	−0.9348
Ectonucleotide pyrophosphatase/phosphodiesterase family member 2	ENPP2	E5RIA2	−0.9187
Ubiquitin carboxyl-terminal hydrolase isozyme L1	UCHL1	P09936	−0.9146
Mitochondrial carnitine/acylcarnitine carrier protein	SLC25A20	O43772	−0.9108
Peptidyl-prolyl cis-trans isomerase FKBP11	FKBP11	Q9NYL4	−0.9097
Phosphoserine aminotransferase	PSAT1	Q9Y617	−0.9057
Delta-1-pyrroline-5-carboxylate synthase	ALDH18A1	P54886	−0.9056

**Table 4:** Proteomic data outlining the effects of hypoxic incubation on hFPT collagen contents as compared to normoxia incubation, presented for each selected collagen type as the corresponding log_2_ FC value. Threshold values for statistical significance determination were specified at −0.9 for downregulation and at 0.9 for upregulation and an FDR significance threshold value was specified at ≤0.01. FC, fold change; FDR, false discovery rate; hFPT, human fetal progenitor tenocytes.

Collagen Types	Log_2_ FC Hypoxia vs. Normoxia
COL1A1	−1.9743
COL12A1	−1.8542
COL1A2	−1.4767
COL3A1	−1.3271
COL4A1	−1.2683
COL5A1	−1.2281
COL5A2	−1.0816
COL14A1	−1.0782
COL18A1	−0.6919
COL11A1	−0.3977
COL6A6	−0.3544
COL16A1	−0.3072
COL6A1	−0.2727
COL6A3	0.1690
COL2A1	0.2588
COL8A1	0.2618

## Data Availability

The data presented in this study are available on request from the corresponding author. The data are not publicly available due to legal and statutory restrictions.

## Supplementary Materials

The following are available online at https://www.mdpi.com/article/10.3390/cells10112872/s1. The “Supplementary Materials” supplementary document contains Figure S1: HIF-1 Western blot whole gel imaging, Figure S2: Collagen 1 Western blot whole gel imaging, and Table S1: Quantitative data of relative HIF-1 detection by Western blot. The “Spreadsheet S1” supplementary document contains the mass spectrometry proteomic dataset analysis. The “Process Parameters” supplementary document contains definitions, targets, parameters, criteria, and gradings relative to the comparative evaluation of both investigated culture conditions. The mass spectrometry proteomics data have been deposited at the ProteomeXchange Consortium via the PRIDE partner repository, with the dataset identifier PXD028359 [78].

## Abbreviations

API	active pharmaceutical ingredient
ASC	adipose-derived stem cells
CD	cluster of differentiation
CHUV	Centre hospitalier universitaire vaudois
CM-FBS	complete medium with FBS
CM-HPL	complete medium with HPL
CPP	critical process parameter
CQA	critical quality attribute
DMEM	Dulbecco’s modified Eagle medium
DTT	dithiothreitol
ECM	extracellular matrix
EPFL	École polytechnique fédérale de Lausanne
ETC	electron transport chain
FACS	fluorescence-activated cell sorting
FASP	filter-aided sample preparation
FBS	fetal bovine serum
FC	fold change
FDR	false discovery rate
FITC	fluorescein isothiocyanate
FSC	forward scatter
GAG	glycosaminoglycan
GMP	good manufacturing practices
GO	gene ontology
HCD	high energy collision dissociation
hFPT	human fetal progenitor tenocytes
HPL	human platelet lysate
IBMX	3-isobutyl-1-methylxanthine
IPC	in-process control
ITS	Insulin–Transferrin–Selenium
KPP	key process parameter
KQA	key quality attribute
MCB	master cell bank
MHC	major histocompatibility complex
MS	mass spectrometry
MSC	mesenchymal stem cell
NCE	normalized collision energy
PBS	phosphate-buffered saline
PCA	principal component analysis
PCB	parental cell bank
PE	phycoerythrin
PPC	post-process control
PRP	platelet-rich plasma
ROS	reactive oxygen species
SD	standard deviation
SDS	sodium dodecyl sulfate
SSC	side scatter
TCA	tricarboxylic acid cycle
TMT	tandem mass tag
USA	United States of America
VitCp	L-Ascorbic acid 2-phosphate sesquimagnesium salt hydrate
WCB	working cell bank

## References

[1] Grognuz A., Scaletta C., Farron A., Raffoul W., Applegate L.A. (2016). Human fetal progenitor tenocytes for regenerative medicine. Cell Transplant..

[2] Grognuz A., Scaletta C., Farron A., Pioletti D.P., Raffoul W., Applegate L.A. (2016). Stability enhancement using hyaluronic acid gels for delivery of human fetal progenitor tenocytes. Cell Med..

[3] Laurent-Applegate L., Grognuz A., Hirt-Burri N., Petrou I.G., Raffoul W. (2014). Cell therapies for tendons: Old cell choice for modern innovation. Swiss Med. Wkly..

[4] Aeberhard P., Grognuz A., Peneveyre C., McCallin S., Hirt-Burri N., Antons J., Pioletti D., Raffoul W., Applegate L.A. (2020). Efficient decellularization of equine tendon with preserved biomechanical properties and cytocompatibility for human tendon surgery indications. Artif. Organs.

[5] Laurent A., Abdel-Sayed P., Grognuz A., Scaletta C., Hirt-Burri N., Michetti M., Roessingh A.D.B., Raffoul W., Kronen P., Nuss K. (2021). Industrial development of standardized fetal progenitor cell therapy for tendon regenerative medicine: Preliminary safety in xenogeneic transplantation. Biomedicines.

[6] Fujikawa K., Ohtani T., Matsumoto H., Seedhom B.B. (1994). Reconstruction of the extensor apparatus of the knee with the Leeds-Keio ligament. J. Bone Joint Surg. Br..

[7] Laurent A., Lin P., Scaletta C., Hirt-Burri N., Michetti M., Roessingh A.S.D.B., Raffoul W., She B.-R., Applegate L.A. (2020). Bringing safe and standardized cell therapies to industrialized processing for burns and wounds. Front. Bioeng. Biotechnol..

[8] Laurent A., Scaletta C., Michetti M., Hirt-Burri N., Roessingh A.S.D.B., Raffoul W., Applegate L.A. (2020). GMP tiered cell banking of non-enzymatically isolated dermal progenitor fibroblasts for allogenic regenerative medicine. Methods in Molecular Biology.

[9] Grayson W.L., Zhao F., Izadpanah R., Bunnell B., Ma T. (2006). Effects of hypoxia on human mesenchymal stem cell expansion and plasticity in 3D constructs. J. Cell. Physiol..

[10] Chung H.-M., Won C.-H., Sung J.-H. (2009). Responses of adipose-derived stem cells during hypoxia: Enhanced skin-regenerative potential. Expert Opin. Biol. Ther..

[11] Das R., Jahr H., Van Osch G.J., Farrell E. (2010). The role of hypoxia in bone marrow–derived mesenchymal stem cells: Considerations for regenerative medicine approaches. Tissue Eng. Part B Rev..

[12] He J., Genetos D.C., Yellowley C.E., Leach J.K. (2010). Oxygen tension differentially influences osteogenic differentiation of human adipose stem cells in 2D and 3D cultures. J. Cell. Biochem..

[13] Verloop R.E. (2011). Progenitor Cells and Hypoxia in Angiogenesis. Ph.D. Thesis.

[14] Frazier T.P., Gimble J.M., Kheterpal I., Rowan B.G. (2013). Impact of low oxygen on the secretome of human adipose-derived stromal/stem cell primary cultures. Biochimie.

[15] Choi J.R., Pingguan-Murphy B., Abas W.A.B.W., Azmi M.A.N., Omar S.Z., Chua K.H., Safwani W.K.Z.W. (2014). Impact of low oxygen tension on stemness, proliferation and differentiation potential of human adipose-derived stem cells. Biochem. Biophys. Res. Commun..

[16] Galeano-Garces C., Camilleri E., Riester S.M., Dudakovic A., Larson D.R., Qu W., Smith J., Dietz A.B., Im H.-J., Krych A.J. (2017). Molecular validation of chondrogenic differentiation and hypoxia responsiveness of platelet-lysate expanded adipose tissue–derived human mesenchymal stromal cells. Cartilage.

[17] Lee S.C., Kim K.-H., Kim O.-H., Lee S.K., Hong H.-E., Won S.S., Jeon S.-J., Choi B.J., Jeong W., Kim S.-J. (2017). Determination of optimized oxygen partial pressure to maximize the liver regenerative potential of the secretome obtained from adipose-derived stem cells. Stem Cell Res. Ther..

[18] Choi S.-H., Kim M.-Y., Yoon Y.-S., Koh D.-I., Cho S.-Y., Kim K.-S., Hur M.-W. (2019). Hypoxia-induced RelA/p65 derepresses SLC16A3 (MCT4) by downregulating ZBTB7A. Biochim. Biophys. Acta Gene Regul. Mech..

[19] Kumar A., Deep G. (2020). Hypoxia in tumor microenvironment regulates exosome biogenesis: Molecular mechanisms and translational opportunities. Cancer Lett..

[20] Mazzatti D., Lim F.-L., O’Hara A., Wood I.S., Trayhurn P. (2012). A microarray analysis of the hypoxia-induced modulation of gene expression in human adipocytes. Arch. Physiol. Biochem..

[21] D’Alessandro S., Magnavacca A., Perego F., Fumagalli M., SanGiovanni E., Prato M., Dell’Agli M., Basilico N. (2019). Effect of hypoxia on gene expression in cell populations involved in wound healing. BioMed Res. Int..

[22] Lee P., Chandel N.S., Simon M.C. (2020). Cellular adaptation to hypoxia through hypoxia inducible factors and beyond. Nat. Rev. Mol. Cell Biol..

[23] Wheaton W.W., Chandel N.S. (2011). Hypoxia. 2. Hypoxia regulates cellular metabolism. Am. J. Physiol. Physiol..

[24] Kang S., Kim S.-M., Sung J.-H. (2014). Cellular and molecular stimulation of adipose-derived stem cells under hypoxia. Cell Biol. Int..

[25] Beegle J., Lakatos K., Kalomoiris S., Stewart H., Isseroff R.R., Nolta J.A., Fierro F.A. (2015). Hypoxic preconditioning of mesenchymal stromal cells induces metabolic changes, enhances survival, and promotes cell retention in vivo. Stem Cells.

[26] Riis S., Stensballe A., Emmersen J., Pennisi C.P., Birkelund S., Zachar V., Fink T. (2016). Mass spectrometry analysis of adipose-derived stem cells reveals a significant effect of hypoxia on pathways regulating extracellular matrix. Stem Cell Res. Ther..

[27] Safwani W.K.Z.W., Choi J.R., Yong K.W., Ting I., Adenan N.A.M., Pingguan-Murphy B. (2017). Hypoxia enhances the viability, growth and chondrogenic potential of cryopreserved human adipose-derived stem cells. Cryobiology.

[28] Hu X., Yu S.P., Fraser J.L., Lu Z., Ogle M.E., Wang J.-A., Wei L. (2008). Transplantation of hypoxia-preconditioned mesenchymal stem cells improves infarcted heart function via enhanced survival of implanted cells and angiogenesis. J. Thorac. Cardiovasc. Surg..

[29] Abdollahi H., Harris L.J., Zhang P., McIlhenny S., Srinivas V., Tulenko T., DiMuzio P.J. (2011). The role of hypoxia in stem cell differentiation and therapeutics. J. Surg. Res..

[30] Kim W.-S., Sung J.-H. (2012). Hypoxic culturing enhances the wound-healing potential of adipose-derived stem cells. Adv. Wound Care.

[31] Lee W.Y., Lui P.P.Y., Rui Y.F. (2012). Hypoxia-mediated efficient expansion of human tendon-derived stem cells in vitro. Tissue Eng. Part A.

[32] Haque N., Rahman M.T., Abu Kasim N.H., Alabsi A. (2013). Hypoxic culture conditions as a solution for mesenchymal stem cell based regenerative therapy. Sci. World J..

[33] Yamamoto Y., Fujita M., Tanaka Y., Kojima I., Kanatani Y., Ishihara M., Tachibana S. (2013). Low oxygen tension enhances proliferation and maintains stemness of adipose tissue-derived stromal cells. BioResearch Open Access.

[34] Hsiao S., Dilley R.J., Dusting G.J., Lim S.Y. (2014). Ischemic preconditioning for cell-based therapy and tissue engineering. Pharmacol. Ther..

[35] Korski K.I., Kubli D.A., Wang B.J., Khalafalla F.G., Monsanto M.M., Firouzi F., Echeagaray O.H., Kim T., Adamson R.M., Dembitsky W.P. (2019). Hypoxia prevents mitochondrial dysfunction and senescence in human c-Kit+ cardiac progenitor cells. Stem Cells.

[36] Zhang Y., Wang B., Zhang W.J., Zhou G., Cao Y., Liu W. (2009). Enhanced proliferation capacity of porcine tenocytes in low O_2_ tension culture. Biotechnol. Lett..

[37] Martin-Rendon E., Hale S.J., Ryan D., Baban D., Forde S.P., Roubelakis M.G., Sweeney D., Moukayed M., Harris A.L., Davies K. (2007). Transcriptional profiling of human cord blood CD133+ and cultured bone marrow mesenchymal stem cells in response to hypoxia. Stem Cells.

[38] Fu H., Luo F., Yang L., Wu W., Liu X. (2010). Hypoxia stimulates the expression of macrophage migration inhibitory factor in human vascular smooth muscle cells via HIF-1α dependent pathway. BMC Cell Biol..

[39] Tello D., Balsa E., Acosta-Iborra B., Fuertes-Yebra E., Elorza A., Ordóñez Á., Corral-Escariz M., Soro I., López-Bernardo E., Perales-Clemente E. (2011). Induction of the mitochondrial NDUFA4L2 protein by HIF-1α decreases oxygen consumption by inhibiting complex I activity. Cell Metab..

[40] Semenza G.L. (2013). HIF-1 mediates metabolic responses to intratumoral hypoxia and oncogenic mutations. J. Clin. Investig..

[41] Dengler V.L., Galbraith M., Espinosa J.M. (2014). Transcriptional regulation by hypoxia inducible factors. Crit. Rev. Biochem. Mol. Biol..

[42] Morotti M., Bridges E., Valli A., Choudhry H., Sheldon H., Wigfield S., Gray N., Zois C.E., Grimm F., Jones D. (2019). Hypoxia-induced switch in SNAT2/SLC38A2 regulation generates endocrine resistance in breast cancer. Proc. Natl. Acad. Sci. USA.

[43] Thangarajah H., Vial I.N., Chang E., El-Ftesi S., Januszyk M., Chang E.I., Paterno J., Neofytou E., Longaker M.T., Gurtner G.C. (2009). IFATS collection: Adipose stromal cells adopt a proangiogenic phenotype under the influence of hypoxia. Stem Cells.

[44] Wang J.-A., He A., Hu X., Jiang Y., Sun Y., Jiang J., Gui C., Wang Y., Chen H. (2009). Anoxic preconditioning: A way to enhance the cardioprotection of mesenchymal stem cells. Int. J. Cardiol..

[45] Zhao F., Grayson W.L., Ma T., Irsigler A. (2009). Perfusion affects the tissue developmental patterns of human mesenchymal stem cells in 3D scaffolds. J. Cell. Physiol..

[46] Huang T.-F., Yew T.-L., Chiang E.-R., Ma H.-L., Hsu C.-Y., Hsu S.-H., Hsu Y.-T., Hung S.-C. (2013). Mesenchymal stem cells from a hypoxic culture improve and engraft achilles tendon repair. Am. J. Sports Med..

[47] Koziel A., Jarmuszkiewicz W. (2017). Hypoxia and aerobic metabolism adaptations of human endothelial cells. Pflugers Arch..

[48] Laurent A., Hirt-Burri N., Scaletta C., Michetti M., Roessingh A.S.D.B., Raffoul W., Applegate L.A. (2020). Holistic approach of Swiss fetal progenitor cell banking: Optimizing safe and sustainable substrates for regenerative medicine and biotechnology. Front. Bioeng. Biotechnol..

[49] Wiśniewski J.R., Zougman A., Nagaraj N., Mann M. (2009). Universal sample preparation method for proteome analysis. Nat. Methods.

[50] Kulak N., Pichler G., Paron I., Nagaraj N., Mann M. (2014). Minimal, encapsulated proteomic-sample processing applied to copy-number estimation in eukaryotic cells. Nat. Methods.

[51] Dorfer V., Pichler P., Stranzl T., Stadlmann J., Taus T., Winkler S., Mechtler K. (2014). MS Amanda, a universal identification algorithm optimized for high accuracy tandem mass spectra. J. Proteome Res..

[52] Kong A.T., LePrevost F.V., Avtonomov D.M., Mellacheruvu D., Nesvizhskii A.I. (2017). MS Fragger: Ultrafast and comprehensive peptide identification in mass spectrometry–based proteomics. Nat. Methods.

[53] R Core Team The R Project for Statistical Computing. https://www.r-project.org/.

[54] Plubell D., Wilmarth P.A., Zhao Y., Fenton A.M., Minnier J., Reddy A.P., Klimek J., Yang X., David L.L., Pamir N. (2017). Extended multiplexing of tandem mass tags (TMT) labeling reveals age and high fat diet specific proteome changes in mouse epididymal adipose tissue. Mol. Cell. Proteom..

[55] Robinson M.D., McCarthy D.J., Smyth G.K. (2010). edgeR: A bioconductor package for differential expression analysis of digital gene expression data. Bioinformatics.

[56] Ritchie M.E., Phipson B., Wu D., Hu Y., Law C.W., Shi W., Smyth G.K. (2015). Limma powers differential expression analyses for RNA-sequencing and microarray studies. Nucleic Acids Res..

[57] Benjamini Y., Hochberg Y. (1995). Controlling the false discovery rate: A practical and powerful approach to multiple testing. J. R. Stat. Soc. Ser. B.

[58] Klopfenstein D.V., Zhang L., Pedersen B.S., Ramírez F., Vesztrocy A.W., Naldi A., Mungall C.J., Yunes J.M., Botvinnik O., Weigel M. (2018). GOATOOLS: A python library for gene ontology analyses. Sci. Rep..

[59] Uchida T., Rossignol F., Matthay M.A., Mounier R., Couette S., Clottes E., Clerici C. (2004). Prolonged hypoxia differentially regulates hypoxia-inducible factor (HIF)-1α and HIF-2α expression in lung epithelial cells. J. Biol. Chem..

[60] Kim J.-W., Tchernyshyov I., Semenza G.L., Dang C.V. (2006). HIF-1-mediated expression of pyruvate dehydrogenase kinase: A metabolic switch required for cellular adaptation to hypoxia. Cell Metab..

[61] Wood S.M., Wiesener M.S., Yeates K.M., Okada N., Pugh C., Maxwell P.H., Ratcliffe P. (1998). Selection and analysis of a mutant cell line defective in the hypoxia-inducible factor-1 α-subunit (HIF-1α). J. Biol. Chem..

[62] Kim S.Y., Choi J.S., Park C., Jeong J.W. (2010). Ethyl pyruvate stabilizes hypoxia-inducible factor 1 alpha via stimulation of the TCA cycle. Cancer Let..

[63] Zheng J., Zhang M., Weng H. (2018). Induction of the mitochondrial NDUFA4L2 protein by HIF-1a regulates heart regeneration by promoting the survival of cardiac stem cell. Biochem. Biophys. Res. Commun..

[64] Smits V.A., Cabrera E., Freire R., Gillespie D. (2018). Claspin—Checkpoint adaptor and DNA replication factor. FEBS J..

[65] Li N., Lam W.H., Zhai Y., Cheng J., Cheng E., Zhao Y., Gao N., Tye B.-K. (2018). Structure of the origin recognition complex bound to DNA replication origin. Nat. Cell Biol..

[66] Nikonova A.S., Astsaturov I., Serebriiskii I.G., Dunbrack R., Golemis E. (2013). Aurora A kinase (AURKA) in normal and pathological cell division. Cell. Mol. Life Sci..

[67] Fabbro M., Zhou B.-B., Takahashi M., Sarcevic B., Lal P., Graham M., Gabrielli B., Robinson P.J., Nigg E., Ono Y. (2005). Cdk1/Erk2- and Plk1-dependent phosphorylation of a centrosome protein, Cep55, is required for its recruitment to midbody and cytokinesis. Dev. Cell.

[68] Xie M., Bu Y. (2019). SKA2/FAM33A: A novel gene implicated in cell cycle, tumorigenesis, and psychiatric disorders. Genes Dis..

[69] Nakamura K., Sakaue H., Nishizawa A., Matsuki Y., Gomi H., Watanabe E., Hiramatsua R., Tamamori-Adachi M., Kitajima S., Noda T. (2008). PDK1 regulates cell proliferation and cell cycle progression through control of cyclin D1 and p27Kip1 expression. J. Biol. Chem..

[70] Politis P.K., Makri G., Thomaidou D., Geissen M., Rohrer H., Matsas R. (2007). BM88/CEND1 coordinates cell cycle exit and differentiation of neuronal precursors. Proc. Natl. Acad. Sci. USA.

[71] O’Brien M. (2007). Structure and metabolism of tendons. Scand. J. Med. Sci. Sports.

[72] Thorpe C.T., Screen H.R.C. (2016). Tendon structure and composition. Adv. Exp. Med. Biol..

[73] Vetro S.W., Bellanti J.A. (1989). Fetal and neonatal immunoincompetence. Fetal Diagn. Ther..

[74] Zhang J., Wang J.H.-C. (2013). Human tendon stem cells better maintain their stemness in hypoxic culture conditions. PLoS ONE.

[75] D’Ippolito G., Diabira S., Howard G.A., Roos B.A., Schiller P.C. (2006). Low oxygen tension inhibits osteogenic differentiation and enhances stemness of human MIAMI cells. Bone.

[76] Kabat M., Bobkov I., Kumar S., Grumet M. (2020). Trends in mesenchymal stem cell clinical trials 2004–2018: Is efficacy optimal in a narrow dose range?. Stem Cells Transl. Med..

[77] Rezakhani L., Kelishadrokhi A.F., Soleimanizadeh A., Rahmati S. (2021). Mesenchymal stem cell (MSC)-derived exosomes as a cell-free therapy for patients infected with COVID-19: Real opportunities and range of promises. Chem. Phys. Lipids.

[78] Perez-Riverol Y., Csordas A., Bai J., Bernal-Llinares M., Hewapathirana S., Kundu D.J., Inuganti A., Griss J., Mayer G., Eisenacher M. (2019). The PRIDE database and related tools and resources in 2019: Improving support for quantification data. Nucleic Acids Res..

[79] World Medical Association (WMA) (2013). Declaration of helsinki: Ethical principles for medical research involving human subjects. JAMA.

