# Supplementary material for: Hypoxic Incubation Conditions for Optimized Manufacture of Tenocyte-Based Active Pharmaceutical Ingredients of Homologous Standardized Transplant Products in Tendon Regenerative Medicine
Source: Cells. 2021 Oct 25;10(11):2872. doi: 10.3390/cells10112872 (PMC8616528; doi:10.3390/cells10112872)
Supplement: Supplementary file 1 [file cells-10-02872-s001.zip › Supplementary Materials/Process Parameters.pdf]

Process Parameters

Contents presented in this Supplementary Document are relative to the process parameters or quality attributes taken into account for the comparison of normoxic (i.e., 5% CO_2; 21% O_2) and hypoxic (i.e., 5% CO_2; 2% O_2) incubation conditions of hFPTs. Specific comparative evaluation results are presented for each parameter or attribute, for the incubation conditions investigated in this study. The objective was to assess if a switch from normoxic toward hypoxic incubation conditions may be considered for hFPT cultures, based on parametric and pondered evaluation results.

Table PP1. Definition of process parameters and quality attributes within the comparative hFPT manufacturing process. For each item, the predefined targets and the IPCs or PPCs to be appropriately monitored are listed, along with the corresponding acceptance criteria. Grading was performed for the comparative evaluation of incubation conditions using the abbreviated nomenclature presented hereafter. (–) = negative effect of hypoxic incubation, (+/-) = no impact of hypoxic incubation, (+) = positive effect of hypoxic incubation. CPPs were defined as parameters exerting a critical effect on the safety and quality of the final manufactured cell batch. KPPs were defined as parameters exerting a key effect on the quality of the final manufactured cell batch. CQAs were defined as attributes presenting critical implications in the safety and quality of the final manufactured cell batch. KQAs were defined as attributes presenting key implications in the quality of the final manufactured cell batch. CPP, critical process parameter; CQA, critical quality attribute; hFPT, human fetal progenitor tenocytes; IPC, in-process control; KQA, key quality attribute; KPP, key process parameter; PPC, post-process control.

**CPP/CQA**<br>**KPP/KQA**	**Targets**	**IPCs/PPCs**	**Acceptance criteria** (cumulative)	**Evaluation of hypoxia conditions vs. standard normoxia conditions**
**CPP/CQA**<br>Cellular morphology	Specific hFPT cellular morphology and behavior maintenance in hypoxic culture<br><br>Absence of observable multiple cell populations in hypoxic culture	Visual monitoring of hFPT cultures during expansion<br><br>Photographic recording of hFPT cultures	Spindle-shaped cells<br><br>Distinctive fibroblastic phenotype<br><br>Absence of multiple cell populations	+/-
**KPP/KQA**<br>Cellular proliferation rate	Maintenance or amelioration of hFPT proliferation rates in hypoxic culture	Evolutive monitoring of population doubling values<br><br>Evolutive monitoring of population doubling times	Equal or superior population doubling values in hypoxia vs. normoxia<br><br>Equal or inferior population doubling times in hypoxia vs. normoxia	+

**KPP/KQA** Cell size distribution	Maintenance of hFPT size distribution in hypoxic culture or relative shift of cell size distribution toward smaller cells	Visual monitoring of hFPT cultures during expansion<br><br>Photographic recording of hFPT cultures<br><br>Determination of cell size distribution by flow cytometry	Maintained cell size distribution or shift of cell size distribution toward smaller cells	+
**CPP/CQA** Cell surface markers	Maintenance of hFPT cell surface marker panel in hypoxic culture conditions	Determination of cell surface marker panel by flow cytometry	Maintenance of cell surface marker panel	+/-
**CPP/CQA** Phenotype plasticity under induction	Maintenance of low hFPT phenotype plasticity in hypoxic culture conditions	Determination of hFPT susceptibility toward chemical adipogenic and osteogenic induction	Maintenance of low cell phenotype plasticity<br><br>Absence of increased susceptibility toward chemical adipogenic and osteogenic induction	+/-
**KPP/KQA** Protein contents	Maintenance of expression for characteristic hFPT protein components in hypoxic culture conditions<br><br>Increase in expression of proteins with potential therapeutic functions in hypoxic culture conditions	Determination of protein expression and protein contents during and following cell expansion	Maintenance of expression for characteristic protein components (e.g., extracellular matrix components)	+

**CPP/CQA** Absence of contaminants	No increased rates of contaminating pathogens detected in retention samples from hypoxic culture conditions	Monitoring of culture medium coloration (i.e., phenol red indicator) and turbidity<br><br>Specific testing for bacteria, fungi, viruses, endotoxins, mycoplasma	Absence of detection for specified contaminants or values of detection < to specified thresholds<br><br>Absence of detection for non-specified contaminants	**+/-**
**CPP/CQA** Cellular viability	Maintenance of adequate cellular viability upon hFPT enzymatic harvest and upon initiation from cryopreservation	Determination of relative cellular viability following cell harvest or cell initiation	Relative cellular viability ≥ 85% after harvest<br><br>Relative cellular viability ≥ 80% after initiation	**+/-**
**CPP/CQA** Cell adherence in culture	Adherence of ≥ 70% of cells after the first 24 h of hypoxic incubation following hFPT cell seeding	Visual monitoring of cultures after 24 h of incubation<br><br>Photographic recording of hFPT cultures	Cell adherence ≥ 70% after 24 h of incubation	**+/-**
**KPP/KQA** Cell monolayer homogeneity	Homogenous growth of hFPT cell monolayer over available culture vessel surfaces in hypoxic culture conditions	Visual monitoring of cell monolayer during expansion<br><br>Photographic recording of cultures	Absence of unpopulated culture surfaces ≥ 15% of the total available culture surface in each vessel	**+/-**
**CPP/CQA** Cell population purity	Specific hFPT cellular morphology and behavior maintenance in hypoxic culture conditions<br><br>Absence of multiple cell populations in culture	Visual monitoring of cultures during expansion<br><br>Photographic recording of cultures<br><br>Determination of cell surface marker panel by flow cytometry	Spindle-shaped cells<br><br>Distinctive fibroblastic phenotype<br><br>Absence of multiple cell populations<br><br>Consistent cell surface marker panel	**+/-**

**KPP/KQA** Cellular metabolism	No modification in hFPT cellular metabolism rate in hypoxic culture conditions	Monitoring of the culture medium coloration (i.e., phenol red indicator) and turbidity	No significant differences in culture medium color and turbidity	**+/-**
**KPP/KQA** Culture confluency evolution	Confirmation of positive hFPT cell monolayer confluency evolution between successive medium exchange procedures	Visual monitoring of confluency at medium exchange procedures<br><br>Photographic recording of cultures	(Confluency value at medium exchange X) ≥ (Confluency value at medium exchange X-1)	**+/-**
**KPP/KQA** Cell type lifespan	No modifications in hFPT cell type lifespan or increased cell type lifespan in hypoxic incubation conditions	In vitro cell type lifespan determination<br><br>Evolutive monitoring of cell population doubling values and doubling times	No modifications in cell type lifespan or increased cell type lifespan	**+**
**KPP/KQA** Total culture period	Conserved or shortened hFPT total culture period for specific in vitro expansions in hypoxic culture conditions	Evolutive monitoring of cell culture periods, from seeding to harvest	Conserved or shortened total culture period for specific in vitro expansions	**+**
