# Supplementary material for: Hypoxic Incubation Conditions for Optimized Manufacture of Tenocyte-Based Active Pharmaceutical Ingredients of Homologous Standardized Transplant Products in Tendon Regenerative Medicine
Source: Cells. 2021 Oct 25;10(11):2872. doi: 10.3390/cells10112872 (PMC8616528; doi:10.3390/cells10112872)
Supplement: Supplementary file 1 [file cells-10-02872-s001.zip › Supplementary Materials/Supplementary Materials.pdf]

Supplementary Materials

Contents presented in this document are relative to acquisition of gel photographic records following Western blot analyses and related data quantification.

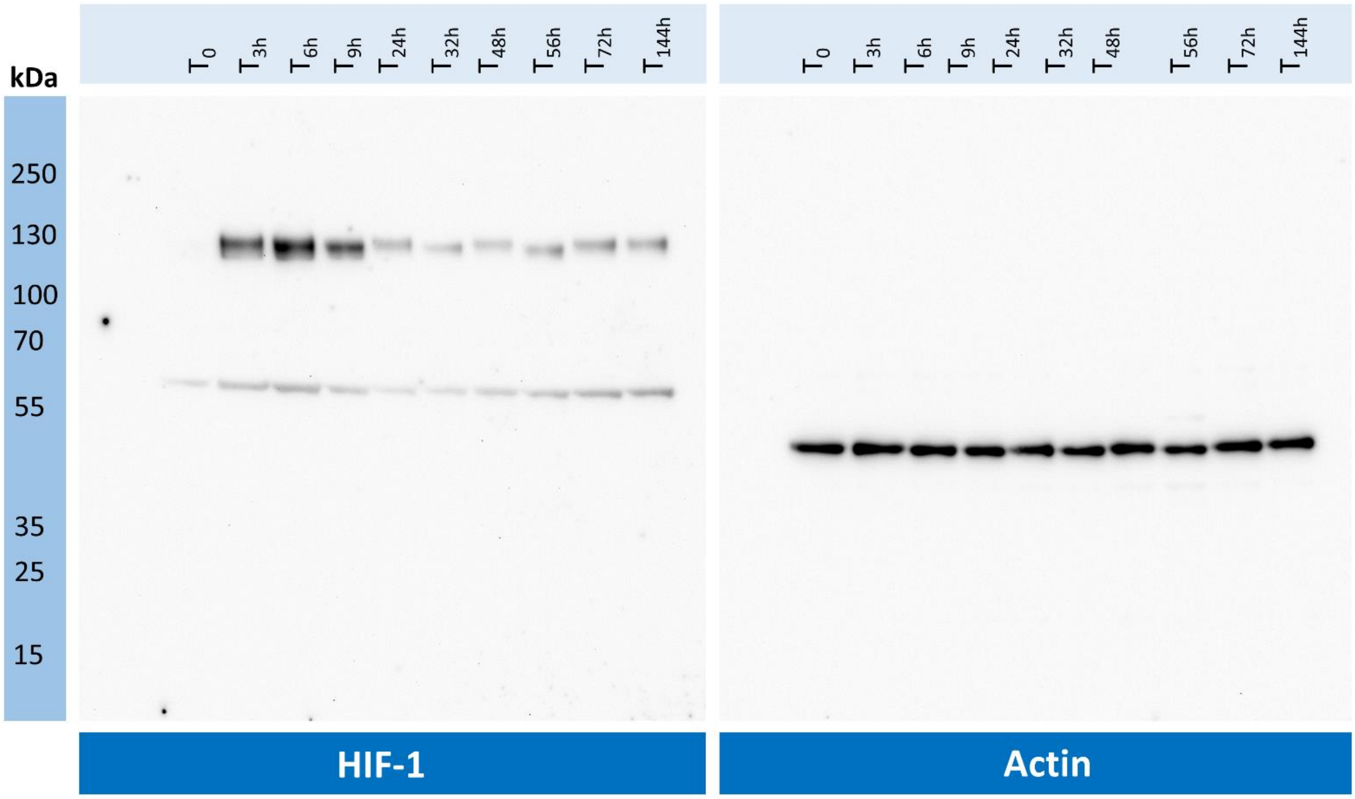

Figure S1. Whole gel photographic records of the timecourse specific analysis of HIF-1α induction upon hFPT exposure to hypoxia incubation conditions, as presented in Figure 1. hFPT, human fetal progenitor tenocytes; kDa, kiloDaltons.

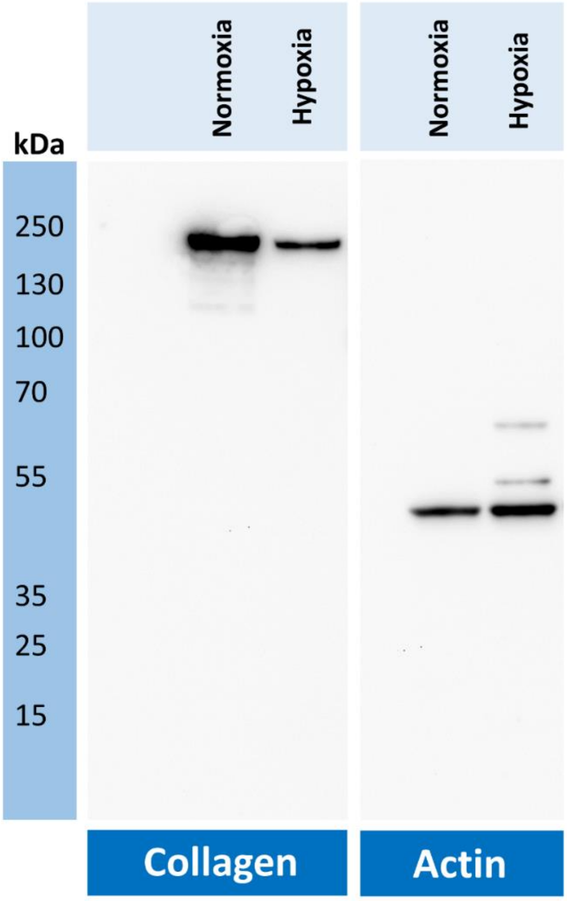

Figure S2. Whole gel photographic records of the comparative data relative to specific hFPT lysate collagen contents in the normoxia incubation conditions and in the hypoxia incubation conditions, as presented in Figure 8. hFPT, human fetal progenitor tenocytes; kDa, kiloDaltons.

Table S1. Quantitative data of relative HIF-1α detection by Western blot, corresponding to ImageJ analysis of the gel presented in Figure 1 / S1.

**Experimental timepoint (h)**	**Ratio of net band values (HIF-1/Actin)**
0	0.023
3	0.600
6	0.862
9	0.580
24	0.193
32	0.121
49	0.116
56	0.214
72	0.212
144	0.230
